# A qualitative exploration of cervical and breast cancer stigma in Karnataka, India

**DOI:** 10.1186/s12905-017-0407-x

**Published:** 2017-08-02

**Authors:** Laura Nyblade, Melissa Stockton, Sandra Travasso, Suneeta Krishnan

**Affiliations:** 1 RTI International, 701 13th ST NW, Suite, Washington, DC 750 USA; 2 Research Triangle Institute Global India Private Limited, 21 Nehru Place, Paharpur Business Centre, Suite no. 610, Nehru Place, India

**Keywords:** Cervical cancer, Breast cancer, Stigma, Psychosocial barriers

## Abstract

**Background:**

Breast and cervical cancer are two of the most common cancers among women worldwide and were the two leading causes of cancer related death for women in India in 2013. While it is recognized that psychosocial and cultural factors influence access to education, prevention, screening and treatment, the role of stigma related to these two cancers has received limited attention.

**Methods:**

Two qualitative exploratory studies. One focusing on cervical cancer, the other on breast cancer, were conducted in Karnataka, India using in-depth interviews and focus group discussions. In the breast cancer study, 59 in-depth interviews were conducted with patients, primary caregivers and healthcare providers. In the cervical cancer study, 147 respondents were interviewed including older and younger women, husbands, healthcare providers and community leaders. While stigma was not the focus of either study, themes relating to stigma emerged and are the focus of this analysis.

**Results:**

Cancer stigma emerged as a general theme across both data sets. It appeared throughout the transcripts as descriptions of how women with breast or cervical cancer would be treated and talked about by husbands, family and the community (manifestations of stigma) and the reasons for this behavior. Stigma as a theme also arose through discussions around managing disclosure of a cancer diagnosis. Stigma was juxtaposed with a narrative of support for women with cancer. Three major themes emerged as driving the manifestations of cancer stigma: fear of casual transmission of cancer; personal responsibility for having caused cancer, and; belief in and fear of the inevitability of disability and death with a cancer diagnosis. Manifestations of cancer stigma were described in terms of experienced (enacted) stigma, including isolation or verbal stigma, and anticipated (fear of) stigma, should a cancer diagnosis be disclosed.

**Conclusions:**

The presence in these communities of cancer stigma and its many forms emerged across both the cervical and breast cancer data sets. Stigma was a feared outcome of a cancer diagnosis and described as a barrier to screening, early diagnosis and treatment seeking for women with symptoms. While further research on cancer stigma is needed, this exploration of some of the driving factors provides insight for future programmatic efforts to reduce cancer stigma and improve access to information, screening and treatment.

**Electronic supplementary material:**

The online version of this article (doi:10.1186/s12905-017-0407-x) contains supplementary material, which is available to authorized users.

## Background

Breast and cervical cancer are two of the most common cancers among women worldwide [1, 2]. Much of the global burden of these two cancers is in low and middle income countries (LMICs) where around 53% of global breast cancer cases and around 85% of cervical cancer cases occur [1, 3]. In India, cervical and breast cancer were the two leading causes of cancer related deaths for women in 2013 [2]; of the estimated 326,300 female cancer deaths, 21.5% were caused by breast cancer and 20.7% were caused by cervical cancer [4]. India, where over 120,000 new cases of cervical cancer are diagnosed annually, bears about a fifth of the global burden of cervical cancer [5]. In 2015, there were an estimated 155,000 new cases of breast cancer and about 76,000 Indian women were estimated to have died of the disease [6, 7]. By 2020, it is estimated that there will be nearly 180,000 new cases of breast cancer and 105,000 new cases of cervical cancer in India [8]. Research has shown that many factors acting at each of the socio-ecological levels – individual, family, community, healthcare facility, and policy – impact access to cervical and breast cancer prevention, screening, treatment, and ultimately health outcomes. These factors include, but are not limited to socioeconomic disparities, healthcare systems, gender inequalities, low levels of knowledge, fear and psychosocial barriers [9–13].

Stigma is increasingly recognized as a critical psychosocial barrier to, and key social determinant of, health [14], its negative impact on health most clearly documented in the field of HIV [15–21]. Recognition of the potential role of stigma to similarly undermine the cancer care continuum [22], particularly cervical and breast cancer prevention, screening, and treatment [10, 11, 13, 23, 24], is beginning to grow, though empirical evidence is still limited. The importance of recognizing and exploring cancer related stigma in India is also gaining attention [13, 22, 24]. For example, a qualitative study conducted in India exploring stigma related to any cancer found that participants believed others would think that cancer was a result of “sin,” that they may rejected by either or both their communities and families, and that they would be isolated due to the false perception of cancer being an infectious disease [22]. A review on underutilization of cervical cancer prevention services in LMICs, including India, identified stigma attached to discussing reproductive health issues as a barrier to knowledge of cervical cancer and its prevention [11]. A qualitative study on challenges to cancer treatment in India identified cultural values and stigma as key barriers to treatment [10], while a qualitative study in Thailand found that women faced social stigma and isolation following breast cancer treatment [25]. Another qualitative study conducted in India found that social stigma as a result of believing breast health problems were a reflection of poor character contributed to hiding breast cancer symptoms [26]. Furthermore, the link between cervical cancer and the human papillomavirus, a common sexually transmitted infection, has further stigmatized this disease in some places [27].

**Fig. 1:**
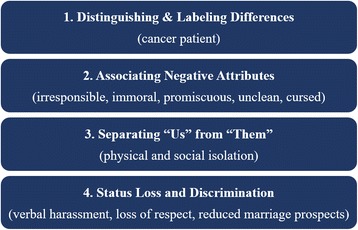
Stigmatization Process. Legend: Sources: Link, B.G. and J.C. Phelan 2001. “Conceptualizing Stigma.” Annual Review of Sociology: 363–385

Stigma is a powerful social process occurring within the context of power that has been described by Link and Phelan as beginning with labelling, followed by stereotyping, which leads to separation, and ultimately results in status loss and discrimination (the endpoint of the stigmatization process) (Fig. 1) [28]. Discrimination is the unfair and unjust action toward an individual or group on the basis of real or perceived status or attributes [29]. Individuals face stigma for differences relating to both health status (e.g. disease specific) and non-health characteristics (e.g. poverty, gender). Individuals or groups with several stigmatized conditions or characteristics often experience multiple intersections of stigma, intensifying the negative effects of stigma [30–33]. Stigma manifests in many ways and can be categorized into several distinct types [34, 35], (Table 1), all of which can lead to negative health and social outcomes for breast and cervical cancer patients and survivors.

**Table 1:** Types of Stigma

Experienced	Stigma that is enacted through interpersonal acts of discrimination
Perceived	Perception of the prevalence of stigmatizing attitudes in the community or among other groups (e.g. healthcare providers)
Anticipated	Fear of stigma, whether or not it is actually experienced
Secondary	Stigma by association, extended to family or other caregivers of stigmatized individual
Observed	Stigma occurring to others that is witnessed or heard about
Layered	The intersecting of stigmas faced by individuals who are part of multiple marginalized groups

Adapted from: Nyblade et al. “Perceived, anticipated and experienced stigma: exploring manifestations and implications for young people’s sexual and reproductive health and access to care in North-Western Tanzania” [35]

Understanding the different types and forms of stigma, as well as underlying causes is necessary to design effective responses to stigma. The need for further investment to understand and respond to cervical and breast cancer stigma has been recognized [23]. To date, few studies have attempted to unpack stigma related to cervical or breast cancer, especially with respect to examining programmatically actionable causes of that stigma. In response, this paper examines stigma, with a focus on causes and manifestations in two exploratory qualitative studies conducted in Karnataka, India. One on breast cancer, the other on cervical cancer.

## Methods

This paper presents analysis of data from two qualitative, exploratory studies, one focusing on breast cancer and the other on cervical cancer. Both studies were guided by the social-ecological model [36–38] as it applies to the cancer care continuum. This approach posits that illness-related behaviours such as care-seeking and experiences are influenced not only by individual characteristics, perceptions, and resources, but also by contextual factors and processes, such as family support, health care organization, and community norms. Zapka et al. (2010) have used this model to understand factors associated with follow-up of abnormal screening test results for breast, cervical and colorectal cancers in the United States, and to guide the identification and evaluation of multilevel interventions to improve follow-up [36]. In our application of this framework, we hypothesized that factors at the level of the individual, family, provider/health care organization, and community will influence breast and cervical cancer prevention, management and survivorship. In keeping with this framework, we examined the drivers and manifestations of cancer stigma at the individual, family, institutional (health providers) and broader community levels.

Both studies were conducted in the southern Indian state of Karnataka between November 2013 and May 2014. While investigating stigma was not the main objective of either study, themes relating to stigma emerged consistently across both studies and are the focus of analysis for this paper. The breast cancer study was conducted among patients and their caregivers at a tertiary care hospital, the cervical cancer study was conducted among women and men in a community setting. Individual descriptions of each study’s setting, participants, data collection methods and data analysis software can be found in Table 2. Sample size for both studies was structured to reach saturation within each of the types of respondents identified as key to interview for the objectives of the study. Convenience sampling within the purposively selected groups was employed for both studies. Interviewers underwent in-depth training on cervical and breast cancers, etiology, prevention, management and consequences; they also underwent additional training on ethics and on conducting focus groups and in-depth interviews. Moreover, they were closely supervised during data collection. The training and supervision aimed to minimize potential interviewer biases. The interviewers, who are not authors of this manuscript, are female, have a mix of education ranging from completing secondary school to Masters degrees, more than 5 years of experience and on-the-job training on survey and qualitative research data collection.

**Table 2:** Study and participant details of the Breast and Cervical Cancer Studies

Parameter	Breast Cancer Study		Cervical Cancer Study			
*Location*	Tertiary care hospital: St. Johns Medical College and Hospital, Bangalore, Karnataka		Community based: Bangalore Rural and Chikkaballapura Districts, Karnataka through collaboration with Cancer Care India (CCI), an NGO that provides cervical cancer education, screening, diagnostic and treatment services.			
*Data collection methods*	- In-depth interviews		- Focus Group Discussions (FGD) - In-depth interviews (IDI)			
*Participants*	Inclusion Criteria					
	- Patients with a histo-pathologically confirmed diagnosis of breast cancer with early, advanced and metastatic disease, their caregivers, and healthcare providers - Above age 18 and fluent in English, Kannada, or Tamil		- *FGD inclusion criteria:* Women eligible for screening (30–45 years, 46–60 years) and husbands of women in this age range - *IDI inclusion criteria:* Women in the exposed villages who had undergone screening, frontline health care workers (physicians, nurses and community health workers), and community leaders (community day care workers, teachers, members of village government)			
	Sample size					
	Participants	Number of IDIs	Participants	Number of FDGs	Number of IDIs	Total number of individuals
	Patients	27	Exposed Villages^b^			
	Primary caregivers	22	Women	4		43
			Screened women		6	6
	Healthcare providers	10	Husbands	2		21
			Unexposed Villages^c^			
	Total	59	Younger Women	4		38
			Husbands	2		18
			Healthcare Providers		8^a^	10
			Community Leaders		10^a^	11
			Total	12	24	147
*Data Analysis software*	NVivo 9.2		NVivo 9.2 and Atlas Ti			

^a^Joint interviews with two or more respondents

^b^Exposed: where CCI implemented outreach programs

^c^Unexposed: where CCI planned to conduct outreach programs but had not yet done so

### Breast cancer (BC) study

Research was conducted at the St. John’s Medical College and Hospital (SJMCH) in Bengaluru (Bangalore), the capital city of the southern Indian state of Karnataka. According to the 3-year report of population based cancer registries for 2012–2014, in the Bangalore registry area breast cancer was the most common cancer among women accounting for 27.5% of all female cancers, with a crude incidence rate of 29.3% and an age-adjusted incidence rate of 34.4% [8].

The study was designed to assess breast cancer patients, their primary caregivers and healthcare providers’ understanding of breast cancer, as well as their perspectives on cancer care, care trajectories, and multilevel influences. This included family and community reactions to symptoms and diagnosis, as well as availability and access to different types of healthcare facilities. To ensure a wide range of experiences and to explore various facilitators or barriers to diagnosis and treatment, patients with a histo-pathologically confirmed diagnosis of breast cancer with varying stage of illness were recruited. Potentially eligible and interested patients and caregivers were referred to the research interviewers by the treating surgeon/physician/nurse for eligibility screening and informed consent. Interviews, lasting 45 to 90 min, were conducted privately in a closed counseling or consultation room at the hospital.

### Cervical cancer (CC) study

Data was collected in villages in two rural districts close to Bangalore city, Karnataka. While there is currently no organized cervical cancer screening program in Karnataka, there are several public and private tertiary hospitals (including the regional cancer center in Bangalore), medical colleges and non-governmental organizations (e.g. Cancer Care India-CCI) that offer cervical cancer screening and linkages to diagnostic and treatment services. According to the 3-year cancer registry report, cervical cancer was the second most common cancer among women in the Bangalore registry area, accounting for 12.3% of all female cancers with a crude incidence rate of 13.1% and an age-adjusted incidence rate of 15.3% [30].

The purpose of the study was to assess perceptions of cervical cancer, screening, and gynecological examinations, as well as explore factors that may facilitate or pose barriers to screening or seeking gynecological services. Data were gathered from villages where CCI’s team of doctors, social workers and local community volunteers had conducted education and screening outreach programs – described in this paper as “exposed” - and villages where CCI planned to conduct outreach programs but had not yet done so – described as “unexposed”. As CCI was involved with cancer education and service delivery in these villages, CCI had established a relationship with the community at large. CCI recruited potential participants in the target groups and referred interested individuals to the research team for eligibility screening. Interviews, lasting 45 to 90 min, were conducted privately at a central location in the village – a school or day care center that was made available for the interviews.

#### Ethics statement

Both study protocols were reviewed and approved by the Office of Research Protection, Institutional Review Board, RTI International. For the breast cancer study, additional approval was obtained from St. John’s National Academy of Health Sciences Institutional Ethics Committee. For both studies, the content of the informed consent form (either in English or the local languages), the intent and objectives of the study, as well as risks and benefits, were verbally explained by interviewers to potential participants. Individuals who provided written consent were enrolled. For the breast cancer study a token of appreciation (refreshment, a nutritional supplement and a folder to hold care-related documents costing approximately USD $8) was given to participants to compensate for their time. For both studies, individual participants had no established relationship with the interviewers prior to recruitment.

#### Data collection and analysis

Interviews and discussions were digitally recorded and interviewers took field notes. Digitally recorded interviews and discussions were translated and transcribed into English. A summary of interview guide topics is provided in Table 3. All English transcripts and recordings were independently reviewed for accuracy and completeness. These transcripts were not returned to participants for comment or correction. Transcripts were managed and analyzed using software packages (NVivo 9.2 and Atlas Ti). The analysis approach used a combination of predetermined and derived themes for data coding. Transcripts were first read repeatedly by the respective analysis teams until content familiarity was high and key themes could be summarized. A codebook was then developed using key themes emerging from the data as well as concepts or issues of interest at study outset. To ensure consistency, two members of each respective team double coded a small number of transcripts, compared their results, and further refined the codebooks. The remaining transcripts were coded by one individual from each research team. Coded data were reviewed to examine similarities and differences within each theme and between groups. Participants did not provide feedback on the analysis. Interview guides for both studies, which were developed by the authors and piloted for comprehensibility, are available as Additional files 1 and 2.

**Table 3:** Questionnaire guide details

Breast Cancer Study	Cervical Cancer Study
Knowledge and awareness about breast cancer Pre-diagnosis - Symptoms - Experience seeking care Diagnosis - Experience receiving diagnosis - Personal and family reaction Treatment - Treatment experience - Side effects Perceptions of care Financial impact Family and social relationships - Family and social support - Changes in relationships Survivorship - Strategies for improving the cancer care trajectory - Future plans beyond treatment Provider role and experiences providing cancer care Provider perspectives on improving breast cancer prevention and treatment	Awareness about cervical cancer - Knowledge of: risk factors for cervical, symptoms, prevention, treatability - Sources of information Facilitators and barriers to cervical cancer screening (uterus/pelvic exams) - Knowledge of screening - Comfort going to or talking about screening and cervical cancer symptoms - Reasons for not screening - Willingness to be screened in the absence of symptoms - Family and community support of cervical cancer screening - Potential strategies for facilitating screening - Stigma: Community & family reactions to cancer - Changes in familial relationships - Social isolation - Shame & embarrassment - Differences in treatment of women with different types of cancer

## Results

Cancer stigma emerged as a general theme across both data sets, though most strongly in the transcripts from the cervical cancer study. This was in part a reflection of the design of the two studies, with questions and probes to explore stigma being a specific sub-focus in the interview guides for the cervical cancer study, but not for the breast cancer study. Within the cervical cancer study, no significant differences in stigma emerged between women who lived in villages that had been exposed to cervical cancer screening versus those living in villages that had not been exposed to screening. Of note is that stigma emerged as a consistent theme within the breast cancer study data even though there were no specific questions on it. Stigma appeared throughout the transcripts as descriptions of how women with breast or cervical cancer would be treated and talked about by husbands, family and the community (manifestations of stigma) and the reasons for this behaviour (drivers). Stigma also emerged through discussion around disclosure of a cancer diagnosis and the pros and cons of sharing that diagnosis, sometimes even with the patient. At the same time, a strong narrative of support for women with cancer also emerged, sometimes with both support and manifestations of stigma expressed in the same transcript, reflecting the nuances of the situation.

**Fig. 2:**
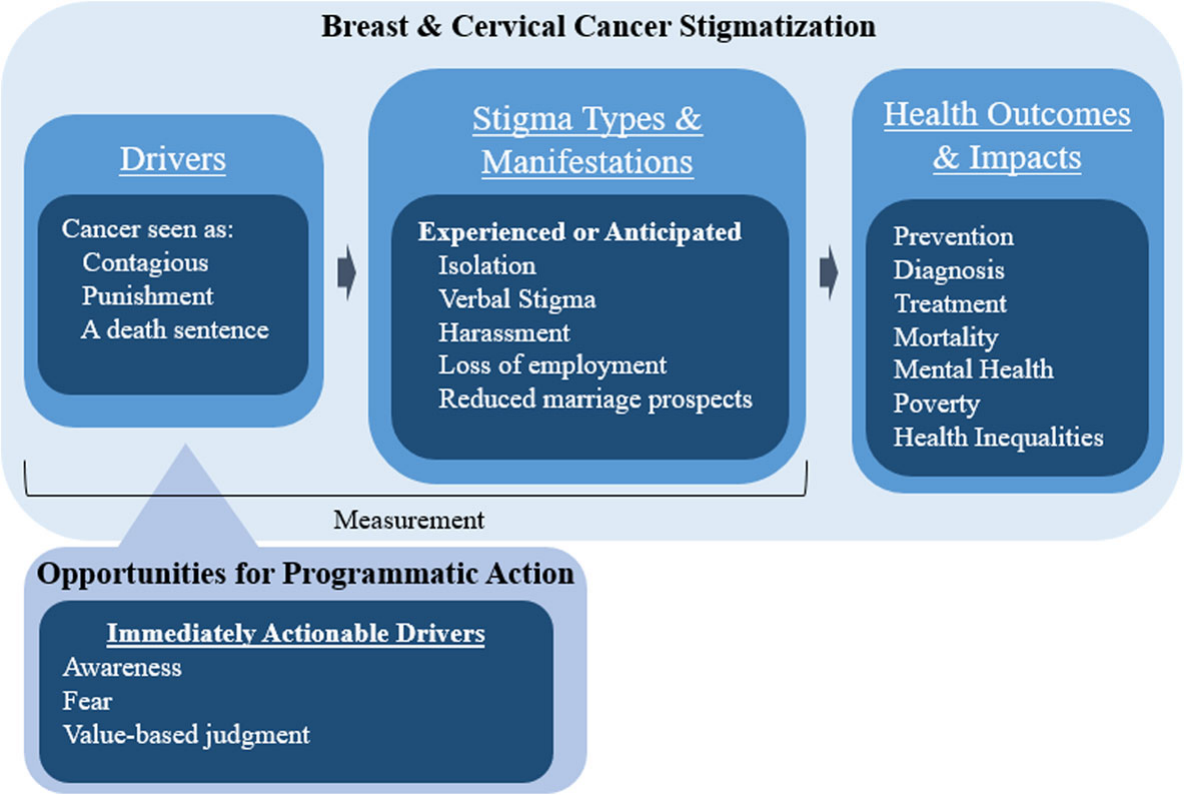
Results Framework

With an eye to entry points for programmatic action to address stigma for improved health outcomes, this section is organized with a focus on the drivers (causes) of stigma that emerged, the manifestations of stigma described, and the consequences that follow stigma. The HIV stigma experience has demonstrated that understanding key immediately actionable drivers of stigma provides a pathway to potential opportunities for programmatic action to reduce stigma. In the case of HIV, stigma reduction has been possible specifically through addressing awareness of stigma, fear, and value-based judgements [39, 40]. Figure 2 illustrates the drivers, manifestations and outcomes of cancer stigma as they emerged in these data sets for breast and cervical cancer stigma. Figure 2 also highlights potential opportunities for programmatic action to reduce cancer stigma that are directly linked to the drivers.

### Drivers

Three major themes emerged as driving the manifestations of cancer stigma described by participants: fear of cancer transmission; personal responsibility for having caused cancer, and; belief in and fear of the inevitability of disability and death with a cancer diagnosis.

#### Cancer as contagious

The fear that cancer could be transmitted through casual contact was the most persistent theme emerging from both data sets, often linked to discussions around physical isolation of people living with cancer and as the reason for doing so: *“once they know that the person is having cancer, they will never let them near. They do not behave normally…. they are afraid that the disease will come to them. They feel the cancer might spread from one person to another*.” (IDI CC healthcare provider**)** There was fear that cancer could be transmitted through sharing eating utensils, sleeping in the same room, playing with children, through cooking food or just general socializing. The quote below typifies the way in which this fear was discussed throughout the transcripts.

*R2: They will avoid going to their places thinking it will spread to them also.*

*R3: If they offer anything to eat they don’t take it. Even they will advise their kids to avoid them.*

*R4: They wouldn’t like to share food in the same plate.*

*R1: They think that they are having cancer and if we eat in their plate, we will also get it. From us it will spread to our family members like this they fear.*

*R2: They think it is contagious disease.*

*R3: All diseases will spread from one person to other.* (CC FG women).

While many respondents noted that cancer was not contagious, and isolating people with cancer was wrong, they also observed that it was still a common belief, that ‘others’ continued to fear they could contract cancer through daily interactions as *“they might feel that they will get the disease if they touch them,”* and thus behaved poorly towards people: “*they don’t care for them.”* (IDI CC Community Leader) As this woman who had been screened for cervical cancer and a healthcare provider from the breast cancer study explain:

*“Everyone mainly have a feeling that it is a contagious disease… The moment they know a person is having cancer then the first thing that comes to their mind is it is a contagious disease… They stay away from the patients. They avoid them in the family. They behave in such a way that the patients leave the house voluntarily. Yes. They do that. But it is actually wrong.”* (IDI CC woman)

*“People are worried that this patient who is on treatment, she will spread the cancer to the children in the house and to the neighbors. So we have seen sadly many times that these patients are unnecessarily isolated because of the wrong notion that these people have done something so wrong to get this disease or they may spread it to people in the house. So there is a lot of stigma associated with cancer. Cancer is a disease in our country which will always be associated with stigma even though it is no fault of the patient.”* (BC healthcare provider)

This fear of cancer being transmissible even extends to those living with cancer. As a breast cancer patient explained, she herself was afraid she could transmit cancer and only after “*I asked doctors and they advised not to fear and told that this is not contagious, even if you eat in the same plate other person will not get this, so it will not spread and you can move with all, without fear. Only after his words I got convinced and started to mingle happily.”* (IDI BC patient) While another respondent living with breast cancer described her continued doubts about whether her cancer was transmissible: *Because relatives think that the disease will spread to them, while eating, talking, they have that kind of fear; even I am unaware whether this will spread like that.”* (IDI BC patient).

The negative impact of this fear and the isolation that ensues on women living with cancer, including being afraid to share their diagnosis, is described by this respondent, also living with breast cancer.

“*Other people think they should not share food, clothes and they should not share soap and also they won’t sleep with others. They should be separate only…I suffered very much and I thought I should not live, that is very difficult for me.”* (IDI BC patient)

Others also recognized the negative impact of the stigmatizing avoidance behaviors on people living with cancer, including how it could lead to self-isolation to avoid the stigma and even suicide.

*“Each and every person will talk bad of her and due to this, she feels she is being isolated. Since others talk badly they feel like staying away from others. They do not mingle with others or go out to functions.”* (IDI CC woman)

#### Cancer as punishment

The belief that a cancer diagnosis in general is the result of having done something bad emerged as a consistent theme in both data sets: “*When a woman is having cancer then they will tell she must have done something wrong and hence she is having cancer.”* (FDG CC women) The supposed bad deed may have occurred in the current, or a past life as explained by these two men in a focus group discussion: “*R1: They will say that it is the reaction of their bad deeds…R2: They will say it is their result of previous births bad deeds.”* (FDG CC men) Belief in cancer as divine retribution for that bad act, as karmic justice, a curse, or the will of god is explained by a healthcare provider:

“*Karma. They use that word. They say, “it must be their last birth’s karma or present life’s karma”…Second thing is, this family has done something wrong to some other people. They have got it due to karma or some wrong thing that they have done. All those things they will say*.” (IDI CC healthcare provider)

The ‘what’ of the assumed bad deed was generally not specified: *“In our community people say that your family got in a curse, that is why disease will be getting in your family itself*.” (IDI BC patient) As this respondent explained, even among educated persons, this general sense of cancer as punishment holds forth:

*“But generally what I have heard is that one would get this if they have done something wrong. They say, “You have done something wrong. You are facing punishment for your wrong doings. God is punishing you.” All those things I have heard from my own husband. I have experienced this. The reason why I am telling this is, we are all educated and as per God’s will we are quite well off. But even at this level there are people who think like this.”* (IDI BC caregiver)

In addition to the general belief of punishment of undefined ‘bad’ deeds, a few respondents raised the issue of sexual transgressions being the cause of the cancer. Implying that women with breast or cervical cancer were responsible for the cancer diagnosis because they had engaged in behaviour society considered immoral or sexually deviant.

*“R2: There will be a different people who start thinking that women having problems are having sex with others.”*

*R1: “Yes. Women are afraid what others would think that way about them”* (FGD CC women)

Interestingly, while several respondents mentioned sexual transmission as one of several possible causes of cancer, transmission of a cancer-causing pathogen (e.g. HPV) through sex did not seem to be the underlying reason for linking sex to cancer. Rather it was more the idea of cancer as punishment for the moral transgression caused by ‘improper sexual relations.’

#### Cancer as a death sentence

A strong belief that cancer is incurable and those who have it will die quickly, permeated the transcripts.

*“Life is finished nothing is there after getting cancer….There is a belief among people that people will die if they get cancer. They don’t know there are many types in cancer and cancers are curable.”* (IDI BC patient)

*“The other thing is that everyone has a feeling that a person having cancer will die soon and will not survive for long.”* (IDI community leader)

The assumed inevitability of death coupled with the fear of illness and death also led to avoidance of the person with cancer, both within and outside the family: “*People in the family will know about it, and they* [cancer patient] *will be tired all the time, so they* [family] *might be afraid to be with them.”* (IDI community leader) Discussing reaction in the broader community, this group of women explained that: *“Some feel anyhow she is not going to survive for more days, some feel she is affected with cancer and we should maintain a distance from her.”* (FGD CC women) This same group went on to discuss how distance will be maintained, for example if a woman with cancer tried to join other women socially they “R1: *wouldn’t accept her casually. R2: When she comes there some other people will go from there.”* (FGD CC women) They explained the reasons for this behavior as a combination of fear of transmission, general discomfort with someone who is sick and a fear of death: *“R1: They feel disturbed when they see people like them. R2: Some will fear it is contagious, so they will move away. R3: Some will fear death.”* (FGD CC women).

In addition to a fear of being near someone who was assumed to be dying, was the assumption that the person would no longer be able to fulfil their expected roles in the family and society as that they would be too sick to continue with work. “*They will not be able to do any work at all. She will feel tired…She cannot do any work at all…I have seen myself that they will be in a very bad state.”* (FGD CC men) As these studies were focused on breast and cervical cancer, and hence women, much of the discussion was focused on no longer being able to fulfil roles within the household as wife (sexual partner), mother and daughter-in-law, as opposed to no longer being able to work in formal employment. *“The woman could feel tired and may not be able cook or wash clothes. They have to send kids for schools. When all these things are disturbed, the pressure is on husband, which will affect relations. The problems will be much more when in-laws are at home. She will have to take care of them along with kids and other household responsibilities. That way pressure will build up… If in- laws live with them, then I think it’s even more difficult.”* (IDI CC community leader).

One group of young women described how a cancer diagnosis would mean that the mother-in-law would begin looking for a new wife for her son. *“If it is between mother in law and daughter in law then mothers in law will be thinking to remarry her son.”* (FGD CC women) While another group discussed how husbands would seek out other sexual partners because the woman would no longer be a fit sexual partner, *“some will have another illegitimate relationship.”* (FGD CC women) A health care provider confirmed this is an issue: *“They acknowledge to us that their husband is not coming near them. He is going to someone else and having relations there.”* (IDI CC healthcare provider) Concern that a diagnosis would lead a husband to stray was also raised as reason why women might hesitate to get screened or seek help for symptoms.

*“Even though men send for the test, ladies don’t go because they are scared that if they have disease husband may leave her and go to another lady. So even though they have disease they don’t go for the test, they think that they* [husband] *will not have sexual relationship with them and they will go to other ladies, if they come to know they have disease. Some people don’t tell even if they have stomach ache thinking that people will assume that they have disease, they don’t go for test, so they are scared. Almost all ladies will have that fear.”* (FGD CC women)

While less commonly discussed, some respondents did note that women with cancer would likely no longer be able to work outside the home, either because they were too weak, or because employers would discriminate against them and not allow them to work.

*“R1: They wouldn’t take her for work at all.”*

*R2: First of all they couldn’t work also.*

*R1: They wouldn’t take her for work.*

*R2: Once they come to know about it they wouldn’t take her for work even if she in starting stage.*

*R3: “Main reason is while working in our field if she had some problems we might have to face the consequences. That’s the main attitude.”* (FGD CC women)

*“They will not be able to do the work properly. If they go to the factory then they will have a target to meet. They will not be able to meet the targets and they will not have the strength. So the factory owners will not co-operate them when they know she has this disease. When they do not encourage, the women will find it difficult to cope up.”* (IDI CC community leader).

In addition to a general fear of death and being near someone perceived to be dying, there was a sense conveyed that a person with cancer, since it is assumed they would no longer be fully functioning members of family or society and will die soon, was simply no longer worth paying attention to. *“From the beginning there is an illusion that this disease cannot be cured so, why should we keep in touch with her? Like this they will think.”* (FDG CC men).

### Manifestations of stigma

Drivers and manifestations (forms) of stigma were closely linked in the transcripts, often within the same sentence. Anticipated (fear of) stigma was the most frequently described form, followed by experienced (enacted stigma). The manifestations of stigma, whether anticipated or experienced, grouped broadly into isolation (physical and social) and verbal abuse.

#### Isolation

The most commonly described forms of stigma were physical and social isolation. *“She will not be entertained. They show disgust and people try to keep distance from her …”* (IDI CC community Leader) The anticipation (fear) that stigma would happen once word got out of a cancer diagnosis was a common thread throughout the transcripts: *“They would fear about the reactions of the neighbors.”* (FGD CC women) As described earlier when discussing fear of transmission as a driver of stigma, avoidance manifested through behaviors such as keeping patients in a separate room, providing separate food as well as eating utensils, not allowing the person with cancer to bathe in the same place as their family members, restricting the touching of food (e.g. cooking or serving), no longer visiting people with cancer in their house or avoiding them in public. Fear of isolation was related to isolation both within and beyond the family.

*“They are not sure what the reaction of family members would be. They are afraid that they might be rejected and isolated. They are not sure if they would be encouraged in this matter.”* (IDI CC healthcare provider)

*“R1: They have fear that people will look down up on them if they detect cancer. If they have children, they will fear it could affect their children”*

*R1: Sometimes they are afraid of relatives and what they would talk about them*

*R2: “They feel they will die early and her family will be spoilt.”* (FGD CC men)

Of note was the occasional juxtaposition of describing the physical separation of a person or items they use as something normal and protective to do, with insistence that the person was being treated well. Pointing to a lack of awareness that the isolating behaviour was stigmatizing.

“*These practical things will be kept separate, but there won’t be discrimination for care and empathy.”* (IDI BC caregiver)

*“They will not mingle with her and they will not share food with her. They will not eat in their plate or they do not share the utensils with her. They might keep things separately for her. But the relation will be there.”* (FGD CC men)

This non-recognition that the described avoidance behaviours are indeed stigmatizing and discriminatory, and unnecessary from a transmission standpoint, is illustrated in this exchange between a community leader and the interviewer:

“*They don’t usually ill-treat her. They would definitely treat her well at home… In the households that I know I have come across keeping things separately for them. They keep separate clothes and plates for them in the family…In the house, there are kids and they advise those people to stay away from children until they are cured. They* [Patient] *also understand the situation and stay away from others. They do have a feeling and they try to take preventive measures.”* (IDI CC community leader)

A commonly expressed fear was that isolation would not only extend to the person living with cancer, but to their families (secondary stigma). Damaging the life chances of children, especially the marriage prospects of a daughter.

*“I don’t speak, because I feel that others shouldn’t know that I have this problem…Why to say this unnecessarily to others, they talk it in a different way, they look at me differently. I have a child. I have to do marriage for her, and her education will get spoiled, as she is the only girl, her education will get spoiled and people around us may speak something, they may say that “your mother had and you may also get like that”, so I avoided as it would have been humiliating. My family members are looking at me well, there are no issues… others might see me differently… They will think low about me and they would have avoided me from them and that would affect my daughter’s future life.”* (IDI BC patient)

*“You take our home care team, there are lots of times when family members will tell, ‘Please don’t park the auto right in front.’ They are worried. Now what are they worried about? One is, will somebody else get it? But more importantly, there is a girl to be married in the family…It is not restricted to only the lower socio-economic strata. The guy could be a PhD from Harvard but he might have that fear.”* (IDI BC healthcare provider)

*“If they do not know there is cancer it is ok or else they will say ‘she has cancer, we do not want girl from this family’.”* (IDI CC community leader)

While not frequently mentioned, a particularly severe and feared form of isolation was abandonment by husband or family. Description of abandonment was often caveated, noting whether it happened or not would be dependent on whether it was a ‘good’ family or not.

*“Some of them will leave their wife. Some of them will treat them well. Some of them keep them away saying you have cancer. Don’t come near me, do not talk to me and I will send you away… like this.”* (IDI CC community leader)

*“In our family some of them have given concerns for us. Some family members they have neglected us…Before they will come very often but now it was reduced…They will not support me more. But, I have lot of support for my friends.”* (IDI BC caregiver)

#### Verbal Abuse

In addition to isolation, another commonly described manifestation of stigma throughout the transcripts was verbal abuse towards people living with cancer, which was expressed in varied ways:

- Scolding: *“One woman who has cancer in our village, that lady who died, that time in communities some people scolded her and they separated her from the house”* (IDI BC patient);
- Teasing: “*well, in the society other people will tease her.”* (IDI CC healthcare provider);
- Talking badly about the person: “*They will use abusing words to her*.” **(**FGD CC women);
- Blaming: *“she used to do bad things. And hence she got it. She deserves to suffer.”* (IDI CC community leader).

While few breast cancer patients recounted experiencing verbal abuse, they talked about what they had heard people saying about other cancer patients behind their backs. This led to the assumption that the same was being said about them out of ear shot: “*They look at me in disgusting way…. People are talking behind me ‘she has cancer who will marry her daughter?’ They don’t talk about me* [to my face], *but I heard them talk about other people very badly. So, I think even they talk about me badly…They say that ‘she has got cancer, who will marry her daughter?’”* (IDI BC patient) Another respondent, in discussing how her relatives responded to her breast cancer diagnosis noted that they gave her positive encouragement, but that she also wondered what they were saying behind her back: “*they were telling ‘don’t worry, nothing will be happen.’ I don’t know what they were talking behind me.”* (IDI BC patient).

An additional theme linked to discussions of verbal abuse was that it was predicated on the woman’s past behavior. Some respondents explained that whether a woman living with cancer received verbal abuse would depend on her assumed moral character.

*“Only if the woman has done wrong things in the past they will scold her. Otherwise, they will not scold her. Yes. They will not scold if her behaviour is good. They will speak badly of her only when her behaviour is not good.”* (IDI CC Healthcare provider)

#### Gossip and distrust in confidentiality of medicalproviders

Another form of verbal abuse that permeated the transcripts was gossip and fear that word would spread quickly, thus triggering isolation and verbal abuse:

*“Once they know they have cancer everyone will get to know about it. It is enough if one come to know, then whole village will know that she has cancer. Once they know they gossip among themselves saying ‘what she did to get cancer?’ They speak badly of people getting cancer. They will say that, maybe she has done something wrong, that’s why she has got it or someone in the house has done something wrong and that is why she has cancer.”* (IDI CC community leader)

*“Some might feel if they have such problems and they share it with others then those people might think wrong about her. She fears what others would talk about her.”* (IDI CC healthcare provider)

Linked to fear of gossip and its consequences were descriptions of how gossip spreads in a community and a fear that interacting with the health system could be the light that sparks gossip. This could happen simply because one was seen going to a health center or special camp for cervical cancer screening, but also reflected an expressed lack of trust that health providers would maintain confidentiality.

“*R2: There is a government hospital in the village. If they go for test there, they feel the information will leak out and everyone will come to know about their problems. Hence, they are afraid to get tested at times.”* (FGD CC women)

“*They fear that everyone will come to know about their problem. Some people do not go to the camp because they feel their problems which they would have kept secret would come out.*” (IDI CC community leader)

*“R2: They feel that the doctor will come to know about the disease they have and will tell to others*”

*R3: Due to this, others will come to know about the disease they have. Thinking about all these things, they do not go to the doctor at all*

*R4: “Some of them are afraid of the disease and afraid where others will come to know about it.”* (FGD CC women)

#### Expressions of support

While many forms of stigma were described throughout the transcripts, expressions of support were also common*, “He* [her husband] *might neglect her. But husbands who really love their wife would care for them and understand that she has the disease due to fate and it is not that she has purposefully fallen sick. So nothing can be done and they show more love to their wife.”* (IDI CC woman). Some respondents felt cancer patients would never be on the receiving end of stigma.

*“They will never talk bad about the person. In fact, they will feel for the person that she has got such a bad disease. When a woman is having this uterus cancer then the information about that goes from one woman to other by word of mouth. They will pity her and tell her husband to take her to the doctor and give her proper treatment.”* (FGD CC men)

### Consequences of stigma

#### Disclosure management

Linked to fear of both isolation and verbal abuse were descriptions of individuals and families actively trying to manage and limit disclosure of a cancer diagnosis to ward off gossip and other forms of stigma. Not only does having to actively manage disclosure add stress, it also limits the possibility of support from a broader range of people.

“*Some people might feel ashamed to talk. They feel shy to talk about the cancer their family is having. People start gossiping that the person has got the disease at such a young age and they scold them.”* (IDI CC woman)

*“She will tell to her husband. She will not tell to anyone else… the woman will never tell her problems to anyone outside. She feels that others will think badly of her when she discusses such problems with them. Hence, she will not tell to anyone else…. She won’t tell to her mother-in-law fearing that she might talk bad about her.”* (IDI CC healthcare provider)

Women with breast cancer and their caregivers confirmed this fear and talked about non-disclosure as a coping mechanism to ward off stigma and protect oneself: “*relatives would think that I have cancer and they think bad about it, so I did not tell them. We have to take care of ourselves.*” (IDI BC patient) Another protective strategy revolved around indicating something had been wrong and was now resolved (e.g. lump has been removed) and/or explaining visits to the hospital as being for other medical issues.

*“When I get ready to go the hospital they ask me ‘where are you going?’ I don’t tell that I am going for radiation treatment, I was telling them that I am going for Physio therapy.”* (IDI BC patient)

*“We haven’t told anyone; we have told that she is getting some treatment for cough. We don’t share it with anyone, nobody will help us so why should we share. If we share others may not mingle with her nor my children, thinking that they also might get it…If we don’t tell them that she has cancer everything will be normal.”* (IDI BC caregiver)

#### Care Seeking

Fear of what might happen if a cancer diagnosis was received was also discussed as a barrier to cervical cancer screening. *“Sometimes they are afraid that other members of the family will discriminate against her if she has any problem. These could be the reason for them not to go for uterus exam.*” (IDI CC community leader). Stigma was also described as one cause for late presentation for medical care, even when symptoms were present:

*“Some women hide the changes in their body. Some fear that if the community comes to know about it they will keep her separately, so to avoid all those humiliation women don’t discuss such things freely. Only when it reaches incurable stage they will tell. Usually village women wouldn’t discuss freely their body internal parts problem. They are afraid of the later reaction from the society. They fear that community might maintain some distance. They might ill-treat her, so they hide it…They will keep suffering silently until they can’t bear it and when they disclose it, it will be in irreparable stage. Even in hospitals they will send them away by declaring it cannot be cured anymore*.” (IDI CC community leader)

*“They are a little more aware, they know a breast lump could be a cancer and usually come to us when it is stage II and there are a group of people who know that it could be something like a tumor or a cancer, but they are afraid to go to a doctor because of the stigma attached to the cancer and the diagnosis and treatment implications. They are afraid of these things and they do not come to a doctor. Even educated people, degree holders, teachers, they come in at a later stage because they tend to sit on their tumor for a longer duration for unknown fear. And I think it is the fear of the diagnosis of cancer and the treatment they want to avoid.”* (IDI BC healthcare provider)

Fear of disclosure through gossip and resultant stigma was also linked to challenges with remaining adherent to treatment.

*“But in villages they feel ashamed to tell it is cancer. If some people are undergoing cancer treatment, then they don’t want to disclose. For the same reason, they are not coming to the doctor. Because of the taboo in the families and villages being a small community, once a family has a problem the whole village comes to know about it. Each and every bit of it will be almost exposed. Because of that part many women are not coming.”* (IDI CC healthcare provider)

## Discussion

At the outset, it is important to note that this analysis is based on data from two qualitative studies with purposive samples of patients, providers and community members in Karnataka, India. By delving into the nature of stigma in Karnataka and offering an in-depth, conceptually grounded description of the drivers and manifestations of stigma, we have gone beyond prior research that has primarily pointed to its existence. Although our findings may not be broadly generalizable, the analysis provides a foundation for future quantitative research on the prevalence of cancer stigma and its associations with health outcomes, as well as the development and testing of stigma reduction interventions.

That cancer stigma is present and manifests in many forms in these communities emerged across both the cervical and breast cancer data sets. Respondents consistently described acts of social and physical isolation ranging from no longer inviting someone living with cancer to family or community social events to physical separation of the person’s eating utensils, clothes, or sleeping quarters to abandonment by spouse or family. Verbal stigma ranging from gossip to outright abuse was also described. Similar manifestations are described by a study on breast cancer treatment and social stigma in Thailand [41], a study on attitudes towards breast cancer among South Asian women living the United Kingdom [42], a study on quality of life of women with breast cancer in India [43], a Nigerian study on the psychosocial concerns of women living with breast and cervical cancer [44], and a Kenyan study on stigma related to both cervical cancer and HIV [45].

The prevalence of stigma and the harm it inflicts led to consistent descriptions of the anticipation (fear) of stigma, both for the individual living with cancer and their family. Respondents described how individuals and families would attempt to contain disclosure of the diagnosis to as few people as possible, even within families. While this has the advantage of warding off anticipated stigma, it also reduces the potential sources of support for the individual and their family. Respondents with breast cancer and their caregivers described doing just this. One common strategy was to explain visits to the hospital as being for other illnesses. This same anticipation of stigma was also described as a reason women did not go for cervical cancer screening and put off seeking medical help even as symptoms worsened. Breast cancer healthcare providers confirmed this, noting that patients sometimes delayed seeking treatment for fear of stigma. The negative effect of stigma, whether anticipated, experienced or internalized on delay or avoidance of screening, delayed entry into care and adherence to treatment is well documented for other diseases, in particular HIV [15–21].

In general, diseases that have one or more of the following characteristics lead to stigma: perceived as easily transmitted or whose mode of transmission is not well understood; acquisition thought to be under the control of the individual, and; when the disease is visibly disfiguring and is assumed to lead to an untimely death. What these data illuminate is that in these communities all three of these factors prevail, therefore the strong descriptions of stigma are perhaps unsurprising. Underlying reasons for cancer stigma emerging from the data revolved around: fear of contagion, the belief that cancer is transmissible; belief in personal responsibility for cancer—cancer as retribution for bad deeds in this life or past, and; cancer as incurable and the inevitability of an untimely death from it. Other studies have also linked the stigmatization of breast and cervical cancer to similar underlying factors. A Chinese survey found that nurses attributed at least some blame to breast and cervical cancer patients for their disease [46], while a qualitative study on attitudes towards breast cancer among South Asian women living in the UK revealed the underlying reason for social and physical isolation was fear of transmission [42]. Several studies have noted the connection between cervical cancer and STIs increases the stigmatization of the disease [27, 47–49]. For example, a qualitative Zambian study found that women attribute cervical cancer to being “promiscuous” or sleeping with many men which is described as feeding into the stigmatization of cervical cancer [48].

Respondents most clearly articulated how fear of casual transmission of cancer led to the physical isolation of a person living with cancer. Some recognized that cancer was not transmissible in this manner and that the discriminatory isolation behaviors were wrong, but noted they continued to occur. Others described these discriminatory behaviors while simultaneously declaring that patients living with cancer were not being treated poorly, but were being well cared for. Physical separation behaviors were viewed as normal, as well as protective, and something that the person living with cancer themselves would engage in to protect others. As this respondent explained: *“That* [isolation] *should not be done actually. But even without their knowledge, they would have started to behave that way. They do not do purposefully.”* (IDI CC screened women) Studies on HIV stigma have found similar contradictions. Families describe how they are treating their family member living with HIV in the best way possible and then go on to describe physical isolation occurring as part of that care [50–53]. While the intention is not to harm, the outcome is often damaging.

Therefore, one key recommendation for programmatic action based on this analysis is simply to implement programs that create awareness of what stigma is. What has been learnt in efforts to reduce HIV stigma is that as stigma is often unintentional, a key strategy to address stigma is to simply create awareness of what stigma is, in very concrete terms – how it manifests in actions and verbally, as well as its consequences [54]. A second and similar recommendation to reduce stigma is to address more clearly and consistently the misconceptions around transmission and, in particular, the specific fears and imagined pathways of transmission [39, 55]. Thirdly, and also tied to the awareness and misconceptions recommendations, is addressing negative beliefs – and the resulting blame – that cancer is a punishment for misdeeds either in the current or past life [39, 56, 57]. Lastly, responses to cancer stigma should leverage supportive family or community members. A narrative of family and community support emerged in the data alongside the presence of prevalent and harmful stigma. This support narrative indicates a strong foundation on which to build stigma-reduction efforts.

## Conclusions

The respondents in these two studies clearly articulated that cancer stigma is present in their lives and communities, is a feared outcome of a cancer diagnosis and a barrier to screening, early diagnosis and treatment seeking for women with symptoms. While further research on cancer stigma is needed, this exploration of the driving factors and stigma manifestations provides insights for future programmatic efforts to reduce stigma and improve access to information, screening and treatment. Notably, the causes of cancer stigma described by respondents are similar to what drives stigma around other diseases such as HIV. A growing body of work on HIV stigma measurement and reduction may provide useful lessons in thinking about how to better understand and respond to cancer stigma. As the experiences in addressing HIV stigma are beginning to demonstrate, measuring and understanding both the underlying causes of stigma and the manifestations has the potential to result in evidence-based responses that can have substantial beneficial health impacts.

## Additional files

Additional file 1: Breast Cancer Interview Guides. Description: *The interview guides used in the breast cancer study*. (DOCX 34 kb)

Additional file 2: Cervical Cancer Interview Guides. Description: *The in-depth interview and focus-group discussion guides used in the cervical cancer study*. (DOCX 61 kb)

## Abbreviations

BC: Breast Cancer
CC: Cervical Cancer
CCI: Cancer Care India
FGD: Focus group discussion
HPV: Human papillomavirus
IDI: In-depth interview
LMICs: Low and middle income countries
STI: Sexually transmitted infection
