# Supplementary material for: A qualitative exploration of cervical and breast cancer stigma in Karnataka, India
Source: BMC Womens Health. 2017 Aug 2;17:58. doi: 10.1186/s12905-017-0407-x (PMC5541646; doi:10.1186/s12905-017-0407-x)
Supplement: Supplementary file 1 — Breast Cancer Interview Guides. Description: The interview guides used in the breast cancer study. (DOCX 34 kb) [file 12905_2017_407_MOESM1_ESM.docx]

**Supplementary File: Breast Cancer Interview Guides**

**Contents**

[In-depth Interview Guide: Breast Cancer Survivors 1](#_Toc485553338)

[In-depth Interview Guide: Providers 6](#_Toc485553339)

[In-depth Interview Guide: Relatives 9](#_Toc485553340)

# In-depth Interview Guide: Breast Cancer Survivors

My name is _________ and I work as a researcher for a study on breast cancer at St. John’s Research Institute. Our study aims to understand women’s experiences with breast cancer diagnosis and treatment. The findings from this study will be used to identify ways in which breast cancer services may be improved.

The interview will take about one hour. Your participation is voluntary, and if you are not comfortable with a question you may choose not to answer it. There are no right or wrong answers. All comments, both positive and negative are welcome. We would also like to tape this interview so we make sure that we do not miss anything, and can write it down at a later time. When we write it down, we will not write down your personal information so there will be no way to identify what you have said after the contents of the tape have been written into notes. The tapes will remain in a locked cabinet and only the study staff will have access to the tapes and transcripts. After the analysis is completed all tapes will be destroyed.

The objective of this interview is to understand breast cancer survivors’ experience of cancer. We will be asking some general information about you and then about your diagnosis, treatment and if appropriate, post-treatment experience. We are interested in learning what has helped make your life after your diagnosis easier or more difficult, and what kinds of supports may be needed by breast cancer patients.

**Introduction**: First, I would like to get some background information about you like how old you are, etc. *[Fill the Sociodemographic Form.]*

**Cancer Care Trajectory**: Now, I would like to explore your experiences and understanding about your health, including how you came to be at St. John’s.

1. ***Knowledge/awareness***: I would like to begin by learning more about the time before you were diagnosed with breast cancer and what you knew about breast cancer.
   1. Had you heard about breast cancer? Can you tell me what you heard?
   2. What kinds of words did you and others in your community use to talk about “cancer” and breast cancer? Was the English word used?
   3. How did others in your family and community talk about “cancer” and “breast cancer”?
   4. Why do you think women experience breast cancer?
   5. What do you people in your family and community say about why women experience breast cancer?
   6. How did you react to women who got breast cancer? [If they knew any women…]
   7. How did others in your family or community react to women with breast cancer?
   8. What happened to women in your community who got breast cancer?
2. ***Pre-Diagnosis***: I would like to learn a little more about the time before you got this diagnosis.
   1. What were the first signs or symptoms that you noticed? [probe whether they were systemic signs/symptoms or tumor-specific signs/symptoms; probe circumstances under which symptoms/signs discovered – self exam? Etc.]
   2. How did you feel about these signs/symptoms? *[Probe recognition as related to cancer, perceived seriousness, worry/anxiety, and coping strategies.]*
   3. What did you do about these signs/symptoms?
      1. Did you talk to anyone about it?
      2. If you spoke to someone about these signs/symptoms, how did they react? What did they recommend? *[Probe for each person they may have spoken to.]*
      3. Did you seek the advice of a health care provider – why or why not? What kind of provider(s) did you consult? How did the health care provider react to your signs/symptoms?
   4. How did the decision about whether to seek health care get made?
      1. Who made the decision?
      2. Who was involved in making the decision? Were there any conflicts or disagreements regarding seeking care? [Probe: can you describe those conflicts, tensions or disagreements? Any associated physical or psychological (denial of resources, humiliation, verbal abuse) violence?
      3. What factors influenced the decision – awareness it could be cancer, beliefs re: cancer, cost/money, not knowing where to go – or not having a doctor easily accessible, time, perceived seriousness?
      4. What kinds of advice/treatment did you receive?
   5. What was the length of time between when you noticed these signs/symptoms and when you consulted the first health care provider? Length of time between when you noticed the signs/symptoms and when you received a diagnosis of breast cancer?
      1. How many providers did you see before you got a diagnosis of breast cancer?
      2. About how much money would you have spent till you got a diagnosis?
3. ***Diagnosis***: Let’s begin by talking about your diagnosis.
   1. When did you receive a diagnosis of breast cancer – did the doctor do a test to confirm the diagnosis? *[Note date when possible – or approximate date Explore if “diagnosis” was histopathologically confirmed or based on signs/symptoms.] 🡪 can explore Pre-Diagnosis period here.*
   2. Where did you first receive this diagnosis? *[Probe type of health facility (private or public? Level?); type of health care provider (physician, nurse, etc)]*
      1. *Can you describe how the diagnosis was made? What kinds of tests were done – did you understand what was happening? How did you feel?*
      2. Who was the information first provided to? *[Probe disclosure, reactions of individual who received information, and respondent’s feelings about disclosure.]*
      3. What additional information was provided at that time? – what did the provider recommend for you?
      4. What else did the doctor offer to help you cope with the diagnosis? – did they offer to link you to treatment? Were treatment options described? Any information on cost and financial impact of the treatment, etc.?
   3. Can you talk a little about how you felt at the moment you received this diagnosis? Your family’s reactions?
      1. Did you think it could be treated? What did you think would be the result of the recommendations your doctor provided?
      2. Did you follow the recommendations? [Probe what happened next?]
4. ***Treatment***: Thank you for taking the time to describe your diagnosis and the time before your diagnosis. Now, I’d like to ask you a few questions about your treatment.
   1. What type of treatment did you receive (chemotherapy, radiotherapy, surgery)? *[NOTE: If patient has undergone all three, choose the most recent treatment to discuss.]*
   2. How long was the treatment? Are you still undergoing treatment?
   3. What did you think the treatment process would be like before you started it?
      1. How do you feel about it now? Is it what you expected?
      2. Are you satisfied with it thus far?
      3. Did your doctors provide you with enough information?
      4. Is there anything you would change about how the doctors gave you/your family information about your treatment? What could your doctor have told you to help better prepare you for treatment?
      5. What might impact/did impact your ability or willingness to complete recommended treatment? [Would need to establish whether the participants had fully completed treatment and where currently in follow-up.]
   4. What were your families’ perceptions of breast cancer and its treatment?
      1. Did your doctors speak with your family? Did they feel informed?
      2. Who are the family members that are living with you now? Are there other family members who are involved in your daily life but not living with you?
   5. What was the date of start of treatment?
      1. Where have you received your treatment? [Probe: public or private?]
      2. How was choice made? By whom?
   6. What was the length of time between when you received a diagnosis and when you started treatment?
      1. What influenced this gap – made it less or more?

*Probes:*

- - - 1. *Expense of transportation to and from the cancer center*
      2. *Repeated hospital visits*
      3. *Cost of treatment*
      4. *Waiting times & other challenges at treatment center (need for bystander, lack of food, etc.)*
      5. *Burden on relatives/lack of relatives who can accompany to treatment. Relatives did not support receiving the treatment.*
      6. *Need to get back to employment/family duties*
      7. *Side effects from the treatments*
      8. *Concern about ability/effectiveness of the treatments to fight the cancer*
      9. *Did not think the treatment was working/mistrust of the Center*
      10. *Fear of community judgment and social stigma*
      11. *Lack of understanding of treatment or treatment complexity*
    1. What made it easier for you to get/start treatment?

*Probes:*

- - - 1. *Family, friends and provider support*
      2. *Hospital/health care providers*
      3. *Insurance*
      4. *Community organization*
      5. *Support group*
    1. How easy or difficult has it been for you to stay and finish your treatment? *[Explore above probes – and if similar barriers and facilitators.]*
    2. Were there any conflicts or disagreements within your family about your treatment? *[Probe for any conflicts, tensions or disagreements? Any associated physical or psychological (denial of resources, humiliation, verbal abuse) violence?*
  1. What kinds of treatment side effects have you experienced?
     1. Were you prepared for them?
     2. Did you lose your hair at any point? How did you feel about that?
  2. Were you given the opportunity to undertake a reconstructive surgery if this was an option? Did you decide to this option? Why (yes/no)?
  3. Had you heard about other women with breast cancer? Did they undergo treatment? Why/why not? What was the result of their treatment?

1. ***Perceptions of health care***
   1. Can you talk about your experience with your cancer treatment provider?
   2. Do you trust your doctor’s opinion?
   3. Were you able to ask your doctors questions about the care you were receiving? How did you feel about that (being able to ask or not being able to ask)?
   4. How is this experience – with the doctors giving you treatment – compared to your usual experience with a doctor?
   5. Since diagnosis have you had to pay from your pocket for medicines, lab tests, treatment or other cancer related service?
      1. Approximately how much would you have paid for your treatment?
      2. How have you dealt with those expenses?
      3. Have these expenses affected the economic situation of your family (including your own situation)?
2. ***Family relationships***
3. Have you talked about your diagnosis with members of your family? Whom? Are there individuals you’ve consciously not shared this information with? *[Probe: why?]*
4. Who in your family has helped care for you? How?
5. Has your husband participated in your care since the diagnosis?
6. Have you noticed changes in your relationship with him?
7. Have your relationships with other members of your family changed? How?
8. How have your family relationships made you feel?
9. Do you think you need or have needed more support from your husband, family, or friends? What type of help?
   - 1. Do you think support groups or voluntary groups (NGOs) could help you?
10. ***Survivorship***
    1. What services [in the community or in the Center] would have been helpful as you and your family prepared for treatment and follow-up?
    2. What did you like about the Center? What did you not like? Where you satisfied with the care you received overall? What could they have done differently to improve your experience?
    3. Do you plan to seek alternative treatments (including ayurveda, homeopathy, spiritual healing and others) instead of/or in conjunction with the recommended treatment? Why?
    4. Any other feedback on how the process of diagnosing and treating breast cancer can be improved?
    5. What advice would you give patients who were diagnosed with breast cancer and were about to initiate treatment?
    6. Can you describe the changes (positive or negative) that you have experienced after diagnosis that affects your daily life? (psychological, family-related, physical, etc.)
       1. Have you returned to work? Do you do any household work?
       2. Have you shared your diagnosis with your family, your friends and others in your community? If no – why not? How have these individuals you’ve disclosed to reacted?
       3. How do people in your community typically view cancer? Breast cancer? *[Probe for reactions, any stigma, embarrassment or shame associated with breast cancer.]*
    7. Looking back since your diagnosis, can you tell me what were the most important moments that you remember?
    8. How do you see your life in the future? Do you see yourself completely recovered?

# In-depth Interview Guide: Providers

My name is _________ and I work as a researcher for a study on breast cancer at St. John’s Research Institute. Our study aims to understand women’s experiences with breast cancer diagnosis and treatment, including health care providers’ perspectives on breast cancer care. The findings from this study will be used to identify ways in which breast cancer services may be improved. The interview will take about one hour. Your participation is voluntary, and if you are not comfortable with a question you may choose not to answer it. There is no right or wrong answers. All comments, both positive and negative are welcome. We would also like to tape this interview so we make sure that we do not miss anything, and can write it down at a later time. When we write it down, we will not write down your personal information so there will be no way to identify what you have said after the contents of the tape have been written into notes. The tapes will remain in a locked cabinet and only the study staff will have access to the tapes and transcripts. After the analysis is completed all tapes will be destroyed.

The objective of this interview is to understand your opinions and experiences related to providing breast cancer care. We will ask some general information about you, followed by questions related to your clinical practice.

**Introduction**: First, I would like to get some background information about you like how old you are, etc. *[Fill the Sociodemographic Form.]*

**Role in Breast Cancer Care Provision**: I would like to begin by learning more about your role in breast cancer provision.

1. Can you describe your breast cancer care-related work?
   1. What do you see as your role in breast cancer prevention and treatment?
2. What aspects of your work do you find fulfilling – think about personal, institutional and patient-related factors that make your work satisfying?
3. What makes your work challenging – again, think about personal, institutional and patient-related factors that make your work difficult to do?
   1. *Probes: For above two questions, explore personal issues: adequacy of training; emotional impact of care; institutional: adequate resources for care provision including space, time, equipment, drugs, policies/support; patient-related: expectations, knowledge, etc.*

**FOR PHYSICIANS/SURGEONS: Cancer Care Trajectory**: Now, I would like to explore your perspectives and practices related to breast cancer diagnosis and treatment.

1. Background
   1. Where do your patients come from? (Geographic area; rural/urban)?
   2. What socio-economic background do they typically have? (Education, income level)?
   3. How do they pay for their health care? (Health insurance? Hospital fund? Etc.)
2. Diagnosis
   1. At what point in the progression of the disease are women typically diagnosed with breast cancer? Break up by %.
   2. Are your patients typically referred from somewhere else or do they on average come to you first?
      1. When patients come to you, do they already have a confirmed diagnosis by the time they are referred to you
      2. ? What kinds of tests/treatments would they have received?
   3. What do you see as the main barriers to ensuring timely diagnosis of breast cancer?
   4. What do you think should ideally happen at the primary care level vs. other higher levels of the system?
   5. How do you think timely diagnosis can be promoted?
3. Treatment
   1. How do you communicate a breast cancer diagnosis? *[Probe – how much information provided? To whom?]*
   2. What are the treatment options that you typically consider?
      1. How and what do you communicate to the patient and/or family members?
      2. To whom do you typically communicate with – patient, family member, both?
      3. Who typically makes decisions about the treatment? Vary by stage of diagnosis?
      4. To what extent does the patient participate in treatment decisions?
   3. What constraints do you/your patients take into account when choosing treatment options?
   4. What are the main factors that influence the timing of treatment initiation – the gap between diagnosis and treatment?
   5. What is the length of survivorship among your patients? (3 or 5-year survival rates?)
4. **FOR ALL CARE PROVIDERS: Cancer Care Experience**: Now, I would like to explore your experiences and observations of breast cancer care.
   1. What are the main challenges you face in treating patients with breast cancer?
   2. What are the main challenges faced by your patients in initiating and completing treatment?

*Probes:*

- - - 1. *Expense of transportation to and from the cancer center*
      2. *Repeated hospital visits*
      3. *Cost of treatment*
      4. *Waiting times & other challenges at treatment center (need for bystander, lack of food, etc.)*
      5. *Burden on relatives/lack of relatives who can accompany to treatment. Relatives did not support receiving the treatment.*
      6. *Need to get back to employment/family duties*
      7. *Side effects from the treatments*
      8. *Concern about ability/effectiveness of the treatments to fight the cancer*
      9. *Did not think the treatment was working/mistrust of the Center*
      10. *Fear of community judgment and social stigma*
      11. *Lack of understanding of treatment or treatment complexity*
    1. What do you do in response to these challenges?
  1. What type of pain mitigation and palliative treatment do you/your facility offer patients? In which cases do you offer pain medication/palliative care?
  2. Does your unit offering any psychosocial or other type of support to help a patient initiate and complete treatment? If yes, please explain.
  3. Can your unit access any funding to subsidize cancer care? If yes, please elaborate.

1. **FOR ALL CARE PROVIDERS: Promoting breast cancer prevention and outcomes**: Now, I would like to explore your opinions regarding breast cancer in India and how to promote prevention and control.
   1. What types of services do you think you should be offered to breast cancer patients?
      1. How might these services be financed?
   2. What types of support do you think breast cancer patients need to improve outcomes?
   3. Do you have any recommendations for how to improve breast cancer prevention and control in India?

# In-depth Interview Guide: Relatives

My name is _________ and I work as a researcher for a study on breast cancer at St. John’s Research Institute. Our study aims to understand women’s experiences with breast cancer diagnosis and treatment. The findings from this study will be used to identify ways in which breast cancer services may be improved.

The interview will take about one hour. Your participation is voluntary, and if you are not comfortable with a question you may choose not to answer it. There are no right or wrong answers. All comments, both positive and negative are welcome. We would also like to tape this interview so we make sure that we do not miss anything, and can write it down at a later time. When we write it down, we will not write down your personal information so there will be no way to identify what you have said after the contents of the tape have been written into notes. The tapes will remain in a locked cabinet and only the study staff will have access to the tapes and transcripts. After the analysis is completed all tapes will be destroyed.

The objective of this interview is to understand your opinions and experiences related to the breast cancer diagnosis and treatment of your relative. We will ask some general information about you and then about your experiences supporting your relatives’ diagnosis, treatment and if appropriate, post-treatment experience. We are interested in learning what has helped make life after breast cancer diagnosis easier or more difficult, and what kinds of supports may be needed by breast cancer patients.

**Introduction**: First, I would like to get some background information about you like how old you are, etc. *[Fill the Sociodemographic Form.]*

**Cancer Care Trajectory**: Now, I would like to explore your experiences and understanding of your relative’s breast cancer diagnosis, including how she came to be at St. John’s.

1. ***Diagnosis***: Let’s begin by talking about the diagnosis.
   1. When did she receive a diagnosis of breast cancer – did the doctor do a test to confirm the diagnosis? *[Note date when possible – or approximate date Explore if “diagnosis” was histopathologically confirmed or based on signs/symptoms.] 🡪 can explore Pre-Diagnosis period here.*
   2. Where did she first receive this diagnosis? *[Probe type of health facility (private or public? Level?); type of health care provider (physician, nurse, etc)]*
      1. *Can you describe how the diagnosis was made? What kinds of tests were done – did you know what was happening? How did you feel?*
      2. Who was the information first provided to? *[Probe disclosure and respondent’s feelings about disclosure.]*
      3. What additional information was provided at that time? What did the doctor/provider recommend?
      4. What else did the doctor offer to help her cope with the diagnosis? Did they offer to link you to treatment? Were treatment options described? Any information on cost and financial impact of the treatment, etc.?
   3. Can you talk a little about how you felt at the moment you heard about her diagnosis?
      1. Had you heard about breast cancer before? What had you heard?
      2. What did you think would be the result of the recommendations the doctor provided?
      3. Were the recommendations followed? [*Probe: what happened next?]*
2. ***Pre-Diagnosis***: I would like to learn a little more about the time before the diagnosis.
   1. Were you aware that your relative had any signs or symptoms? [*probe what signs/symptoms; respondent’s reactions/advice*]
   2. How did the decision to seek health care get made?
      1. Who made the decision?
      2. Who was involved in making the decision? Were there any conflicts or disagreements regarding seeking care? [*Probe: conflicts, tensions or disagreements?]*
      3. What factors influenced the decision – awareness it could be cancer, beliefs re: cancer, cost/money, not knowing where to go – or not having a doctor easily accessible, time, perceived seriousness?
   3. What was the length of time between when your relative noticed these signs/symptoms and she consulted the first health care provider? Length of time between when she noticed the signs/symptoms and when she received a diagnosis of breast cancer?
      1. How many providers did she see before she got a diagnosis of breast cancer?
      2. About how much money would she have spent till she got a diagnosis?
3. ***Treatment***: Thank you for taking the time to describe the diagnosis and the time before your relative’s diagnosis. Now, I’d like to ask you a few questions about the treatment.
   1. What type of treatment has your relative received (chemotherapy, radiotherapy, surgery)? *[NOTE: If patient has undergone all three, choose the most recent treatment to discuss.]*
   2. How long has the treatment been? Is your relative still undergoing treatment?
   3. What did you think the treatment process before it started?
      1. Is it what you expected?
      2. Did the doctors speak with you about it? [Probe: how much information are the doctors giving your relative vs. you/other family members?]
      3. Is there anything you would change about how the doctors gave you/your relative information about the treatment? What could your doctor have told you to help better prepare your relative and you for treatment?

If relative appears to be primary decision-maker, explore following:

- 1. What was the date of start of treatment?
     1. Where have you received your treatment? [Probe: public or private?]
     2. How was choice made? By whom?
  2. What was the length of time between when your relative received a diagnosis and when she started treatment?
     1. What influenced this gap – made it less or more?
        1. *Probes:*
        2. *Expense of transportation to and from the cancer center*
        3. *Repeated hospital visits*
        4. *Cost of treatment*
        5. *Waiting times & other challenges at treatment center (need for bystander, lack of food, etc.)*
        6. *Burden on relatives/lack of relatives who can accompany to treatment. Relatives did not support receiving the treatment.*
        7. *Need to get back to employment/family duties*
        8. *Side effects from the treatments*
        9. *Concern about ability/effectiveness of the treatments to fight the cancer*
        10. *Did not think the treatment was working/mistrust of the Center*
        11. *Fear of community judgment and social stigma*
        12. *Lack of understanding of treatment or treatment complexity*
     2. What made it easier for her to get/start treatment?
        1. *Probes:*
        2. *Family, friends and provider support*
        3. *Hospital/health care providers*
        4. *Insurance*
        5. *Community organization*
        6. *Support group*
     3. How easy or difficult has it been for her to stay and finish her treatment? *[Explore above probes – and if similar barriers and facilitators.]*
     4. Were there any conflicts or disagreements within your family about your treatment?
  3. What has been your role in supporting/taking care of your relative?
     1. Have you faced any difficulties in playing this role?
     2. What may have made it easier for you to play this role?
     3. Is there any support you wish you had?

1. ***Perceptions of health care***
   1. Can you talk about your experience with the cancer treatment provider?
   2. Do you trust the doctor’s opinion?
   3. Were you able to ask the doctors questions about the care your relative is receiving? How did you feel about that (being able to ask or not being able to ask)?
   4. Since diagnosis have you had to pay from your pocket for medicines, lab tests, treatment or other cancer related service?
      1. Approximately how much would you have paid for your treatment?
      2. How have you dealt with those expenses?
      3. Have these expenses affected the economic situation of your family (including your own situation)?
2. ***Family relationships***
3. How has your relationship with your relative changed since her diagnosis?
4. Have you spoken about your relative’s cancer diagnosis with others in the family? If no, why not? If yes, how have they reacted?
5. How have your other family relationships changed?
6. Do you think you need or have needed more support from family or friends? What type of help?
   - 1. Do you think support groups or voluntary groups (NGOs) could help you?
7. ***Survivorship***
   1. What services [in the community or in the Center] would have been helpful as your relative has prepared for treatment and follow-up?
   2. What did you like about the Center? What did you not like? Where you satisfied with the care you received overall? What could they have done differently to improve your experience?
   3. Will your relative seek alternative treatments (including ayurveda, homeopathy, spiritual healing and others) instead of/or in conjunction with the recommended treatment? Why?
   4. Any other feedback on how the process of diagnosing and treating breast cancer can be improved?
   5. What advice would you give family members of patients who were diagnosed with breast cancer?
   6. Can you describe the changes (positive or negative) that you have experienced after your relative’s diagnosis that affects your daily life? (psychological, family-related, physical, etc.)
      1. Has it affected your employment, role in household work?
      2. How do your family, your friends and others in your community view your relative/you?
   7. Looking back since your diagnosis, can you tell me what were the most important moments that you remember?
   8. How do you see your life in the future? Do you see your relative completely recovered?
