# Supplementary material for: A qualitative exploration of cervical and breast cancer stigma in Karnataka, India
Source: BMC Womens Health. 2017 Aug 2;17:58. doi: 10.1186/s12905-017-0407-x (PMC5541646; doi:10.1186/s12905-017-0407-x)
Supplement: Supplementary file 2 — Cervical Cancer Interview Guides. Description: The in-depth interview and focus-group discussion guides used in the cervical cancer study. (DOCX 61 kb) [file 12905_2017_407_MOESM2_ESM.docx]

**Supplementary File: Cervical Cancer Interview Guides**

**Contents**

[Focus Group Guide: Women in communities exposed to screening info 2](#_Toc485553175)

[Focus Group Guide: Husbands 7](#_Toc485553176)

[Focus Group Guide: Women in unexposed to screening group 12](#_Toc485553177)

[In-depth Interview Guide: Community Leaders 17](#_Toc485553178)

[In-depth Interview Guide: Health Care Workers 21](#_Toc485553179)

[In-depth Interview Guide: Women who have undergone cervical cancer screening 26](#_Toc485553180)

# Focus Group Guide: Women in communities exposed to screening info

*(Note: inclusion criteria will be having been exposed to screening info. Women in these communities who have not been exposed to screening info get the same FGD guide as women in unexposed communities.)*

Name of moderator:

Name of note taker:

Date:

Start time:

Duration:

*Notes on moderating FGD:*

1. *The introduction below should not be abbreviated, every sentence is important.*
2. *Use the text below as a script. You can read it aloud, as long as you make eye contact with the participants.*
3. *Enough time needs to be allotted for this introduction so it sets the stage for rules and expectations of FGD.*

**Introduction and Guidelines**

Hello. My name is ___________, and I work for an American organization the Research Triangle Institute, International. As we have explained, we are doing a study on cervical cancer screening.

For this discussion, we would like to learn from you why you think some women go for cervical cancer screening, while some women do not. To facilitate the discussion we will ask you a few questions about knowledge, awareness of and attitudes towards cervical cancer screening among families, relatives and neighbors in your communities.

You are encouraged to participate in the discussion and answer as many questions as possible. Your participation is voluntary and, if you are uncomfortable with a question or some part of the discussion, you may choose not to answer.

We are interested in all of your ideas, comments and suggestions about issues that we will discuss with you. There is no right or wrong answers in this discussion. All comments, both positive and negative are welcome. Please feel free to disagree with one another – we would like to have many points of views so that we can understand a situation from many angles. The only request we have is that every person should share her/his view points with consideration to each another. We would like your open and honest opinion, thoughts, perceptions and views on cervical cancer and screening for it, because in order for us to make recommendations about how to improve cervical cancer awareness level and its prevention, we need to have open and diverse answers. Otherwise, our work will not benefit people appropriately.

We want this to be a group discussion, so you need not wait for me to call on any one of you individually to get an opinion. However, we would like to hear each of you. So, if someone else is speaking, please allow them to finish before you make your point. Please give everyone a chance to talk – so please be brief and to the point. Also please ensure that everyone in the group talks and not just one person carries out the whole discussion.

We would also like to tape this discussion so we make sure that we do not miss anything, and can write it down at a later time. When we write it down, we will not write down your personal information so there will be no way to identify what you have said after the contents of the tape have been written into notes. The tapes will remain in a locked cabinet and only the study staff will have access to the tapes and transcripts. After the analysis is completed all tapes will be destroyed. Please speak one at a time so that the tape recorder can pick up everyone’s comments. All comments are considered confidential and used for research purposes only. We also ask that each of you maintain confidentiality and not share what individuals in this room have said to others outside of this room. Of course you may share this discussion with others – we would only like to request that you may not identify the individuals involved today to maintain privacy and confidentiality.

Do any of you have any questions for me before we begin?

(INSTRUCTION: Take note of questions. Once all questions are answered, proceed)

Introduction: First, I would like to get some background information about you like your age, educational background, marital status etc. [Fill the Socio-demographic Form.]

**Details of the participants**

| Sr No | Age | Education | Marital Status | Occupation |
| --- | --- | --- | --- | --- |
| 1 |  |  |  |  |
| 2 |  |  |  |  |
| 3 |  |  |  |  |
| 4 |  |  |  |  |
| 5 |  |  |  |  |
| 6 |  |  |  |  |
| 7 |  |  |  |  |
| 8 |  |  |  |  |
| 9 |  |  |  |  |
| 10 |  |  |  |  |

AWARENESS ABOUT CERVICAL CANCER

1. Have you ever heard about cancer of the mouth of the uterus (Cervical Cancer)?
   1. If so, what do you know about Cervical Cancer?
   2. How did you get this information? If not specified, ask who they got the information from specifically (mother-in-law, husband, neighbors, health facility etc.)
2. What women do you think are at greatest risk of getting Cervical Cancer (Probe: Specifically about different age groups, specific groups of women)
   1. Why are these women at greatest risk?
3. Can you tell us what are the risk factors or causes of Cervical Cancer?

(Probe: Role of diet, life style (smoking, sexual behavior/number of partners—of woman, husband), family history, a virus, any misconceptions – cancer is infectious, caused to supernatural causes, karmic fruits etc.)

1. Are you aware of any symptoms of cervical cancer?
   1. If, so what are those?
   2. Who told you about these symptoms? [If specific people or other sources (e.g. pamphlet, radio, TV) not provided, Probe: Who specifically told you about these symptoms? (family member, healthcare worker, neighbor, media, other?)]
2. Do you think women in your family and communities are at risk of Cervical Cancer?
   1. If yes, why?
   2. If not, why not?
3. Do you think women can do things to avoid getting cervical cancer?  If so, what can they do?
4. Is there anything a woman can do to find cervical cancer early?
5. Do you think cervical cancer can be treated?
6. Is there anything a woman can do to find cervical cancer early?
7. Can cervical cancer be treated?
8. Who should be tested for cervical cancer?

Facilitators and Barriers to cervical cancer screening

1. What have you heard about methods or ways through which one can detect cervical cancer before there are symptoms?
   1. Who or where did you hear this information?
   2. Do women in your community go for cervical cancer screening exams?
   3. If yes, why do women in your community go? Probe: ( symptoms (what kind of symptoms), knows someone who has been for exam, family member recommends, health care worker encouraged it, want to protect health)
   4. If not, why not?
2. Are women in your family and community comfortable going for cervical cancer screening?
   1. If yes, what makes them comfortable to go?
   2. If no, what makes them uncomfortable?
3. Do women in your community talk with anyone about cervical cancer screening?
   1. If yes, who do they talk to?
   2. If no, why not?
4. What conditions would make it easier for women to go for cervical cancer screening? Probe (female doctors, free screening, more convenient locations, privacy, confidentiality)
5. What are the reasons women in your communities may not go for cervical cancer screening exams, when they are available? **(Probe:** uncomfortable getting examined by male doctor, economic, social, fear of detecting illness (probe what kind of illness), discomfort with screening exam itself, not necessary to go if no symptoms/not needed, long wait time, don’t trust or respect the provider)
6. Do you think women would are willing to go for cervical cancer screening, even when they do not have any problems (symptoms)?
   1. If no, why not?
7. How would you convince a family member or neighbor to go for screening? What would you say?
8. In your community, if a woman has problems like bleeding or abdominal pain, who does she talk to about those problems? (Probe: mothers-in-law, husband, health worker, neighbors)
   1. If the answer is no one, then probe: why do women not talk about these problems?
9. Do you think women feel comfortable talking about problems like bleeding or abdominal pain with their husband?
   1. Why? Why not?
   2. What about other family members? Anyone else women feel comfortable talking to about such problems?
10. In your community, if a woman had problems like bleeding or abdominal pain, how would her husband or other family members respond? Probe: would they support her to go to a medical provider?

STIGMA

1. Do you personally know anyone with cancer? If not, let’s assume a family member or neighbor is suffering with cancer:
2. What are/would be the reactions of other family members and the neighbors to a woman with cancer?
   1. Do they think less of him/her?
   2. Gossip or speak badly about the woman or her family?—what do they say? What words are used to describe the woman?
   3. Would relationships with the woman or her family change? If yes, how?
3. What do you think are the most common problems faced by women with cancer? (*Probe: At home/ in the village or neighborhood/workplace/ socially discriminated*)
4. Do you think women who have cancer are isolated by society? If so, why?  If not, why not?
5. Do you think people talk about cancer freely and openly in your society with one another? What about cancers specific to women, like cervical or breast cancer? (If there is a difference, probe why they think there is a difference)
6. Do you think the family feels ashamed/ embarrassed to tell others if someone in their family is suffering from cancer? *(Probe: Does it affect the relationships with family members)* What about cancers specific to women, like cervical or breast cancer? (If there is a difference, probe why they think there is a difference)
7. Do people feel uncomfortable being around a woman with cancer? *(Probe: Sharing stuff/living with a cancer patient/ living close to a cancer patient/ working with a cancer patient, sitting or eating next to a cancer patient*).
   1. If so, why?  What about if it is cancer of a female organ, like breast or cervical cancer. Does that make people more or less uncomfortable? Why?
   2. Do people worry about "catching" it from another person?
8. Are there differences in the way women with cancer are viewed and treated by their families and the community if they have ‘female’ cancers, that is of the breast, uterus or mouth of the uterus compared to if they had a non-female organ cancer (e.g. brain, lung, blood)?
   1. If yes, why?

I have asked you all the questions I wanted to. Thank you for your time and patience. Do you have any questions for me?

# Focus Group Guide: Husbands

Name of moderator:

Name of note taker:

Date:

Start time:

Duration:

*Notes on moderating FGD:*

1. *The introduction below should not be abbreviated, every sentence is important.*
2. *Use the text below as a script. You can read it aloud, as long as you make eye contact with the participants.*
3. *Enough time needs to be allotted for this introduction so it sets the stage for rules and expectations of FGD.*

**Introduction and Guidelines**

Hello. My name is ___________, and I work for an American organization the Research Triangle Institute, International. As we have explained, we are doing a study on going for tests to check for cancer, in particular female cancers, including cancer of the mouth of the uterus.

For this discussion, we would like to learn from you what your opinions are about a female cancer called cancer of the mouth of the uterus and an exam to test for it. Do not worry if you do not know about this cancer or the test to check for it, we will explain to you shortly about them. To facilitate the discussion we will ask you a few questions about knowledge, awareness of and attitudes towards cancer and testing for cancer for women among husbands, families, relatives and neighbors in your communities.

You are encouraged to participate in the discussion and answer as many questions as possible. Your participation is voluntary and, if you are uncomfortable with a question or some part of the discussion, you may choose not to answer.

We are interested in all of your ideas, comments and suggestions about issues that we will discuss with you. There is no right or wrong answers in this discussion. All comments, both positive and negative are welcome. Please feel free to disagree with one another – we would like to have many points of views so that we can understand a situation from many angles. The only request we have is that every person should share her/his view points with consideration to each another. We would like your open and honest opinion, thoughts, perceptions and views on cancer of the mouth of the uterus and a test to check for it, because in order for us to make recommendations about how to improve awareness prevention of cancer of the mouth of the uterus, we need to have open and diverse answers. Otherwise, our work will not benefit people appropriately.

We want this to be a group discussion, so you need not wait for me to call on any one of you individually to get an opinion. However, we would like to hear each of you. So, if someone else is speaking, please allow them to finish before you make your point. Please give everyone a chance to talk – so please be brief and to the point. Also please ensure that everyone in the group talks and not just one person carries out the whole discussion.

We would also like to tape this discussion so we make sure that we do not miss anything, and can write it down at a later time. When we write it down, we will not write down your personal information so there will be no way to identify what you have said after the contents of the tape have been written into notes. The tapes will remain in a locked cabinet and only the study staff will have access to the tapes and transcripts. After the analysis is completed all tapes will be destroyed. Please speak one at a time so that the tape recorder can pick up everyone’s comments. All comments are considered confidential and used for research purposes only. We also ask that each of you maintain confidentiality and not share what individuals in this room have said to others outside of this room. Of course you may share this discussion with others – we would only like to request that you may not identify the individuals involved today to maintain privacy and confidentiality.

Do any of you have any questions for me before we begin?

(INSTRUCTION: Take note of questions. Once all questions are answered, proceed)

Introduction: First, I would like to get some background information about you like your age, educational background, marital status etc. [Fill the Socio-demographic Form.]

**Details of the participants**

| Sr No | Age | Education | Marital Status | Occupation |
| --- | --- | --- | --- | --- |
| 1 |  |  |  |  |
| 2 |  |  |  |  |
| 3 |  |  |  |  |
| 4 |  |  |  |  |
| 5 |  |  |  |  |
| 6 |  |  |  |  |
| 7 |  |  |  |  |
| 8 |  |  |  |  |
| 9 |  |  |  |  |
| 10 |  |  |  |  |

AWARENESS ABOUT CERVICAL CANCER

1. What words come to your mind when you think of cancer?
2. What do you think are the most common cancers in Indian women?
3. Have you ever heard about cancer of the mouth of the uterus (Cervical Cancer)?
   1. If so, what do you know about Cervical Cancer? How did you get this information?
      1. ***If not specified, PROBE: who did you get the information from? (wife, mother, mother-in-law, neighbors, health facility, community leader, media etc.)***
   2. If not: read the following text to explain about cervical cancer: “Cancer is when the cells in a part of the body grow fast and produce a lot of extra tissue. In women, the most common cancer affects the mouth of the uterus. There are several ways in which a health worker can detect cervical cancer even before there are symptoms. A health worker like a nurse or doctor who has gotten training can do a simple check-up for cancer. In this check-up, they look at the mouth of the uterus, paint some vinegar on it, and see if there are any changes that look like cancer.
4. Which women do you think are at greatest risk of getting cancer of the mouth of the uterus (Cervical Cancer) ***(Probe: Specifically about different age groups, specific groups of women***)
   1. Why are these women at greatest risk?
5. Do you think cervical cancer can be treated?
6. Do you think women in your family and communities are at risk of Cervical Cancer?
   1. If yes, why?
   2. If not, why not?

Facilitators and Barriers to uterus (pelvic) exams

Now I would like to ask you about uterus exams for women.

1. Do you know about uterus exams for women?
   1. If yes, what do you know? ***(Probe, what are they for?)***
      1. Where did you get this information? (Wife, friends, health center, media etc)
   2. If no---read explanatory text below explaining what a uterus exam is: *“*Uterus exams are when a health worker examines a woman’s private parts to check if she is healthy.”
2. Do you think women in your community go for uterus exams?
   1. If yes, why do women in your community go for uterus exams?
      1. If not mentioned, PROBE: pregnancy, symptoms (what kind of symptoms), knows someone who has been for exam, husband encourages, family member recommends, health care worker encouraged it, want to protect reproductive health or health in general to maintain family responsibilities, fears may have cancer
   2. If not, why not?
      1. ***If these issues do not come up, Probe on these issues***: husband not supportive, family not supportive, distance, cost, does not feel comfortable, exam room is not private, fear health provider will share results (or fact that woman has come to exam) with others (not keep confidentiality), fear of exam, fear of cancer
3. Do you think men in this community are comfortable with their wives going for uterus exams?
   1. If yes, what makes them comfortable with their wives going for uterus exam?
   2. If no, what makes them uncomfortable?
4. Do men in this community talk with their wives about uterus exams?
   1. If yes, what do they talk about?
   2. If no, why are uterus exams not talked about between husbands and wives?
5. Do men in this community support and encourage their wives to go for uterus exams?
   1. If yes, why?
      1. What kind of support do they provide? ***(Probe to get all possible types of support, e.g. encouragement, transport, money)***
   2. If no, why not?
6. What conditions would make it easier for men to support women to go for uterus exams?
   1. ***If not discussed, PROBE for: female doctors, free screening, more convenient locations, comfortable place to be examined, place to be examined that is completely private (privacy), confidence that health provider will not share any information with others about the woman coming for a test or the results (confidentiality)***
7. What are the reasons women in your communities may not go for uterus exams, if they are available?
   1. ***If not mentioned, PROBE: uncomfortable getting examined by male doctor, economic, social, fear of detecting illness (probe what kind of illness), discomfort with screening exam itself)***
8. Do you think men would support their wives to go for a uterus exam as part of a routine/regular health check-up, even if the wife is not having have any problems (symptoms)?
   1. If yes, why?
   2. If no, why not?
9. Do you think women would be willing to go for a uterus exam as part of a routine/regular health check-up, even if they are not having have any problems (symptoms)?
   1. If yes, why?
   2. If no, why not?
10. In your community, if a wife has problems like bleeding or abdominal pain, who does she talk to about those problems? (***Probe: mothers-in-law, husband, health worker, neighbors)***
    1. If the answer is no one, then probe: why do women not talk about these problems?
11. Do you think women feel comfortable talking about problems like bleeding or abdominal pain with their husband?
    1. Why? Why not?
    2. What about other family members? Anyone else women feel comfortable talking to about such problems?
12. Do you think men feel comfortable talking about problems like bleeding or abdominal pain with their wives?
    1. Why? Why not?
    2. What about other family members? Anyone else men feel comfortable talking to about such problems if their wife is experiencing them?
13. In your community, if a woman had problems like bleeding or abdominal pain, how would her husband or other family members respond? Probe: would they support her to go to a medical provider?

Check-up for Cervical cancer:

***Read explanatory text***: “As I explained earlier, cancer is when the cells in a part of the body grow fast and produce a lot of extra tissue. In women, the most common cancer affects the mouth of the uterus. There are several ways in which a health worker can detect cervical cancer even before there are symptoms. A health worker like a nurse or doctor who has gotten training can do a simple check-up for cancer. In this check-up, they look at the mouth of the uterus, paint some vinegar on it, and see if there are any changes that look like cancer.

1. If a screening test were available for cervical cancer in your community, do you think husbands would support their wives to go for the test?
   1. If yes, why? ***Probe: Even if they had no symptoms?***
   2. If no, why? What about if they had symptoms? Would that make husbands more likely to support their wives to go for a test?
2. If a screening test were available for cervical cancer in your community, do you think women would be willing to go for the test?
   1. If yes, why? Even if they had no symptoms?
   2. If no, why? What about if they had symptoms? Would that make them more likely to go?
3. If a screening test were available for cervical cancer in your community, how would you convince your wife to go for screening? What would you say?
   1. What about other men, how would you convince them to support their wives to go for a test?

Stigma

1. Do you personally know anyone with cancer? If not, let’s assume a female family member or neighbor is suffering with cancer:
2. What are/would be the reactions of other family members and the neighbors to a woman with cancer?
   1. Do they think less of her?
   2. Gossip or speak badly about the woman or her family?—what do they say? What words are used to describe the woman?
   3. Would relationships with the woman or her family change? If yes, how?
3. What do you think are the most common problems faced by women with cancer?
   1. *Probe: At home/ in the village or neighborhood/workplace?*
   2. *Probe: What types of problems do women with cancer face? ( socially discriminated/not able to access credit/lose customers for a business*)
4. Do you think women who have cancer are isolated by society?
   1. If so, why?
5. Do you think people talk about cancer freely and openly in your society with one another?
   1. What about cancers specific to women, like cervical or breast cancer?
      1. ***If there is a difference, probe: why is there a difference?***
6. Do you think the family feels ashamed/ embarrassed to tell others if a woman in their family is suffering from cancer? *(Probe: Does it affect the relationships with family members)*
   1. What about cancers specific to women, like cervical or breast cancer?
      1. ***If there is a difference, probe: why is there a difference?)***
7. Do people feel uncomfortable being around a woman with cancer?  *(Probe: Sharing stuff/living with a cancer patient/ living close to a cancer patient/ working with a cancer patient, sitting or eating next to a cancer patient*).
   1. If so, why?
   2. Do people worry about "catching" it from another person?
8. Are there differences in the way women with cancer are viewed and treated by their families and the community if they have ‘female’ cancers, that is of the breast, uterus or mouth of the uterus compared to if they had a non-female organ cancer (e.g. brain, lung, blood)?
   1. If yes, why?

I have asked you all the questions I wanted to. Thank you for your time and patience. Do you have any questions for me?

# Focus Group Guide: Women in unexposed to screening group

Name of moderator:

Name of note taker:

Date:

Start time:

Duration:

*Notes on moderating FGD:*

1. *The introduction below should not be abbreviated, every sentence is important.*
2. *Use the text below as a script. You can read it aloud, as long as you make eye contact with the participants.*
3. *Enough time needs to be allotted for this introduction so it sets the stage for rules and expectations of FGD.*

**Introduction and Guidelines**

Hello. My name is ___________, and I work for an American organization the Research Triangle Institute, International. As we have explained, we are doing a study on going for tests to check for cancer, in particular female cancers, including cancer of the mouth of the uterus.

For this discussion, we would like to learn what you might know about female cancers and talk to you in particular about a test to check for cancer of the mouth of the uterus. We will explain more about cancer of the mouth of the uterus and this test in a few minutes. To facilitate the discussion we will ask you a few questions about knowledge, awareness of and attitudes towards cancers, in particular female cancers, among families, relatives and neighbors in your communities.

You are encouraged to participate in the discussion and answer as many questions as possible. Your participation is voluntary and, if you are uncomfortable with a question or some part of the discussion, you may choose not to answer.

We are interested in all of your ideas, comments and suggestions about issues that we will discuss with you. There is no right or wrong answers in this discussion. All comments, both positive and negative are welcome. Please feel free to disagree with one another – we would like to have many points of views so that we can understand a situation from many angles. The only request we have is that every person should share her/his view points with consideration to each another. We would like your open and honest opinion, thoughts, perceptions and views on female cancers and tests to check for cancer of the mouth of the uterus, because in order for us to make recommendations about how to improve awareness and prevention of cancer of the mouth of the uterus, we need to have open and diverse answers. Otherwise, our work will not benefit people appropriately.

We want this to be a group discussion, so you need not wait for me to call on any one of you individually to get an opinion. However, we would like to hear each of you. So, if someone else is speaking, please allow them to finish before you make your point. Please give everyone a chance to talk – so please be brief and to the point. Also please ensure that everyone in the group talks and not just one person carries out the whole discussion.

We would also like to tape this discussion so we make sure that we do not miss anything, and can write it down at a later time. When we write it down, we will not write down your personal information so there will be no way to identify what you have said after the contents of the tape have been written into notes. The tapes will remain in a locked cabinet and only the study staff will have access to the tapes and transcripts. After the analysis is completed all tapes will be destroyed. Please speak one at a time so that the tape recorder can pick up everyone’s comments. All comments are considered confidential and used for research purposes only. We also ask that each of you maintain confidentiality and not share what individuals in this room have said to others outside of this room. Of course you may share this discussion with others – we would only like to request that you may not identify the individuals involved today to maintain privacy and confidentiality.

Do any of you have any questions for me before we begin?

(INSTRUCTION: Take note of questions. Once all questions are answered, proceed)

Introduction: First, I would like to get some background information about you like your age, educational background, marital status etc. [Fill the Socio-demographic Form.]

**Details of the participants**

| Sr No | Age | Education | Marital Status | Occupation |
| --- | --- | --- | --- | --- |
| 1 |  |  |  |  |
| 2 |  |  |  |  |
| 3 |  |  |  |  |
| 4 |  |  |  |  |
| 5 |  |  |  |  |
| 6 |  |  |  |  |
| 7 |  |  |  |  |
| 8 |  |  |  |  |
| 9 |  |  |  |  |
| 10 |  |  |  |  |

AWARENESS ABOUT CERVICAL CANCER

1. What words come to your mind when you think of cancer?
2. What do you think are the most common cancers in Indian women?
3. Have you ever heard about cancer of the mouth of the uterus (Cervical Cancer)?
   1. If so, what do you know about Cervical Cancer? How did you get this information?
      1. ***If not specified Probe: who do women get information from specifically (mother-in-law, husband, neighbors, health facility etc.)***
   2. If not: read the following text to explain about cervical cancer: “Our body is made of cells. Cancer is a disease in which these cells don’t work well, grow fast, and produce a lot of tissue. Cervical cancer is when this kind of growth happens on the mouth of the uterus – also called the cervix.”
4. Which women do you think are at greatest risk of getting Cervical Cancer ***(Probe: Specifically about different age groups, specific groups of women)***
   1. Why are these women at greatest risk?
5. Do you think cervical cancer can be treated?
6. Do you think women in your family and communities are at risk of Cervical Cancer?
   1. If yes, why?
   2. If not, why not?

Facilitators and Barriers to uterus (pelvic) exams

Now I would like to ask you about uterus exams for women. *“*Uterus exams are when a health worker examines a woman’s private parts to check if she is healthy.”

1. Do women in your community go for uterus exams?
   1. If yes, why do women in your community go for uterus exams?
      1. ***If these issues do not come up in the course of the response, Probe on these possible reasons:*** pregnancy, symptoms (what kind of symptoms), knows someone who has been for exam, husband encourages, family member recommends, health care worker encouraged it, want to protect reproductive health or health in general to maintain family responsibilities, fears may have cancer
   2. If not, why not?
      1. ***If these issues do not come up, Probe on these issues***: husband not supportive, family not supportive, distance, cost, does not feel comfortable, exam room is not private, fear health provider will share results (or fact that woman has come to exam) with others (not keep confidentiality), fear of exam, fear of cancer, wait time, don’t trust of respect provider, not needed/necessary.
2. Are women in your family and community comfortable going for uterus exams?
   1. If yes, what makes them comfortable to go?
   2. If no, what makes them uncomfortable?
3. Do women in your community talk with anyone about uterus exams?
   1. If yes, who do they talk to?
   2. If no, why are uterus exams not talked about?
4. What conditions would make it easier for women to go for uterus exams?
   1. ***If these do not come up in the course of the discussions, Probe on these possible reasons:***  female doctors, free screening, more convenient locations, comfortable place to be examined, place to be examined that is completely private (privacy), confidence that health provider will not share any information with others about the woman coming for a test or the results (confidentiality)
5. What are the reasons women in your communities may not go for uterus exams, if they are available?
   1. ***If these do not arise in the course of the discussion, Probe on these reasons: no knowledge, no symptoms, husband not supportive, family not supportive, no clinic close by, money for travel or exam, uncomfortable getting examined by male doctor, economic, social, fear of detecting illness (probe what kind of illness), discomfort with screening exam itself)***
6. Do you think women would be willing to go for a uterus exam as part of a routine/regular health check-up, even when they do not have any problems (symptoms)?
7. In your community, if a woman has problems like bleeding or abdominal pain, who does she talk to about those problems? ***(If not mentioned in the course of the discussion, Probe: what about mothers-in-law, husband, health worker, neighbors)***
   1. If the answer is no one, then probe: why do women not talk about these problems?
8. Do you think women feel comfortable talking about problems like bleeding or abdominal pain with their husband?
   1. Why? Why not?
   2. What about other family members? Anyone else women feel comfortable talking to about such problems?
9. In your community, if a woman had problems like bleeding or abdominal pain, how would her husband or other family members respond? ***If not mentioned in the course of the discussion****,* ***Probe: would they support her to go to a medical provider?***

Cervical cancer screening:

***Read explanatory text***: “As I explained earlier, cancer is when the cells in a part of the body grow fast and produce a lot of extra tissue. In women, the most common cancer affects the mouth of the uterus. There are several ways in which a health worker can detect cervical cancer even before there are symptoms. A health worker like a nurse or doctor who has gotten training can do a simple check-up for cancer. In this check-up, they look at the mouth of the uterus, paint some vinegar on it, and see if there are any changes that look like cancer.

1. If a screening test were available for cervical cancer in your community, do you think women would be willing to go for the test?
   1. If yes, why? ***Probe: Even if they had no symptoms?***
   2. If no, why? What about if they had symptoms? Would that make them more likely to go?
2. If a screening test were available for cervical cancer in your community, how would you convince a family member or neighbor to go for screening? What would you say?

STIGMA

1. Do you personally know anyone with cancer? If not, let’s assume a family member or neighbor is suffering with cancer:
2. What are/would be the reactions of other family members and the neighbors to a woman with cancer?
   1. Do they think less of him/her?
   2. Gossip or speak badly about the woman or her family?—what do they say? What words are used to describe the woman?
   3. Would relationships with the woman or her family change? If yes, how?
3. What do you think are the most common problems faced by women with cancer?
   1. *Probe: At home/ in the village or neighborhood/workplace*
   2. *Probe: what specifically happens to her in the village/neighborhood/workplace? (e.g. socially discriminated, loses job, loses customers, cannot get loans, excluded from village groups, e.g. microfinance groups*)
4. Do you think women who have cancer are isolated by society?
   1. If so, why?
5. Do you think people talk about cancer freely and openly in your society with one another?
   1. What about cancers specific to women, like cervical or breast cancer? (***If there is a difference, PROBE: why do you think there is a difference?)***
6. Do you think the family feels ashamed/ embarrassed to tell others if someone in their family is suffering from cancer? *(****Probe: Does it affect the relationships with family members)***
   1. What about cancers specific to women, like cervical or breast cancer? ***(If there is a difference, PROBE: why do you think there is a difference?)***
7. Do people feel uncomfortable being around a woman with cancer?  ***(Probe if not mentioned: Sharing stuff/living with a cancer patient/ living close to a cancer patient/ working with a cancer patient, sitting or eating next to a cancer patient).***
   1. If so, why?  What about if it is cancer of a female organ, like breast or cervical cancer. Does that make people more or less uncomfortable? Why?
   2. Do people worry about "catching" cancer from another person?
8. Are there differences in the way women with cancer are viewed and treated by their families and the community if they have ‘female’ cancers, that is of the breast, uterus or mouth of the uterus compared to if they had a non-female organ cancer (e.g. brain, lung, blood)?
   1. If yes, why?

I have asked you all the questions I wanted to. Thank you for your time and patience. Do you have any questions for me?

# In-depth Interview Guide: Community Leaders

Hello. My name is ___________, and I work for an American organization (Research Triangle Institute, International). As you know, we are doing a study in Karnataka state on health problems affecting women who are between the ages of 30 and 60 years. The objective of this discussion is to understand awareness of cancer and especially cancers that affect women, and access to and use of health services related to cancers that affect women in your community.

The interview will take about 30-45 minutes. Your participation is voluntary, and if you are not comfortable with a question you may choose not to answer it. There are no right or wrong answers. All comments, both positive and negative are welcome. We would also like to tape this interview so we make sure that we do not miss anything, and can write it down at a later time. When we write it down, we will not write down your personal information so there will be no way to identify what you have said after the contents of the tape have been written into notes. The tapes will remain in a locked cabinet and only the study staff will have access to the tapes and transcripts. After the analysis is completed all tapes will be destroyed.

**Introduction**: First, I would like to get some background information about you like how old you are, etc. *[Fill the Socio-demographic Form.]*

| Age | Education | Marital Status | Occupation/Role in Community |
| --- | --- | --- | --- |
|  |  |  |  |

Awareness about cervical cancer

1. Are there other words to describe “cancer”?
2. What do you think are the most common cancers among Indian women?
3. Have you ever heard about cancer of the mouth of the uterus (“Cervical Cancer”)?
   1. If so, what do you know about Cervical Cancer?
   2. How did you get this information?
      1. ***If not specified, PROBE:*** *who did you get the information from? (wife, mother, mother-in-law, neighbors, health facility, community leader, media etc.)*
   3. ***If not: read the following text to explain about cervical cancer:*** “Our body is made of cells. Cancer is a disease in which these cells don’t work as they should, grow fast, and produce a lot of tissue. Cervical cancer is when this kind of growth happens on the mouth of the uterus – also called the cervix.”
4. Which women do you think are at greatest risk of getting cancer of the mouth of the uterus (Cervical Cancer) ***(Probe:*** *about different age groups, specific groups of women*)
   1. Why are these women at greatest risk?
5. Do you think women in your family and community are at risk for cervical cancer?
   1. If yes, why?
   2. If not, why not?
6. Is there anything a woman can do to find cervical cancer early?
7. Do you think cervical cancer can be treated?

Facilitators and Barriers to uterus (pelvic) exams

Now I would like to ask you about uterus exams for women.

1. Have you heard about uterus exams for women?
   1. If yes, what do you know? ***(Probe, what are they for?)***
      1. Where did you get this information? (Wife, friends, health center, media etc)
   2. If no---read explanatory text below explaining what a uterus exam is: *“*Uterus exams are when a health worker examines a woman’s private parts to check if she is healthy.”
2. Do you think women in your community go for uterus exams?
   1. If yes, why do women in your community go for uterus exams?
      1. If not mentioned, PROBE: pregnancy, symptoms (what kind of symptoms), knows someone who has been for exam, husband encourages, family member recommends, health care worker encouraged it, want to protect reproductive health or health in general to maintain family responsibilities, fears may have cancer
   2. If not, why not?
      1. ***If these issues do not come up, Probe on these issues***: husband not supportive, family not supportive, distance, cost, does not feel comfortable, exam room is not private, fear health provider will share results (or fact that woman has come to exam) with others (not keep confidentiality), fear of exam, fear of cancer
3. Do you think men in this community are comfortable with their wives going for uterus exams?
   1. If yes, what makes them comfortable with their wives going for uterus exam?
   2. If no, what makes them uncomfortable?
4. Do men in this community support and encourage their wives to go for uterus exams?
   1. If yes, why?
      1. What kind of support do they provide? ***(Probe to get all possible types of support, e.g. encouragement, transport, money)***
   2. If no, why not?
5. What conditions would make men more supportive of their wives going for a uterus exam?
   1. ***If not discussed, PROBE for:*** *female doctors, free screening, more convenient locations, comfortable place to be examined, place to be examined that is completely private (privacy), confidence that health provider will not share any information with others about the woman coming for a test or the results (confidentiality)*
6. Do you think women would be willing to go for a uterus exam as part of a routine/regular health check-up, even if they are not having any problems (symptoms)?
   1. If yes, why?
   2. If no, why not?
7. In your role as a ______________ (e.g., panchayat member, self-help group leader, etc.):
   1. Do you or would you encourage women to go for uterus exams?
      1. If yes, what would enable you to encourage women?
      2. If no, why not?
   2. Do you or would you support women to go for a uterus exam?
      1. If yes, what kind of support do you think you could provide?
      2. If no, why not?
8. In your community, if a woman has problems like bleeding or abdominal pain, who do you think she talks to about those problems? (***Probe:*** *mothers-in-law, husband, health worker, doctor, neighbors****)***
   1. If the answer is no one, then probe: why do women not talk about these problems?
9. Do you think women feel comfortable talking about problems like bleeding or abdominal pain with their husband?
   1. Why? Why not?
   2. What about other family members? Anyone else women feel comfortable talking to about such problems?
10. Do you think men feel comfortable talking about problems like bleeding or abdominal pain with their wives?
    1. Why? Why not?
    2. Who else do men talk to if their wife is experiencing these problems?
11. In your community, if a woman had problems like bleeding or abdominal pain, how would her husband or other family members respond? ***Probe***: would they support her to go to a health care provider?
12. What are the reasons women in your communities may not go for uterus exams, if these exams are available?
    1. ***If not mentioned, PROBE:*** *uncomfortable getting examined by male doctor, economic, social, fear of detecting illness (probe what kind of illness), discomfort with screening exam itself.*

Check-up for cervical cancer

***Read explanatory text***: “As I explained earlier, cancer is when the cells in a part of the body grow fast and produce a lot of extra tissue. In women, the most common cancer affects the mouth of the uterus. There are several ways in which a health worker can detect cervical cancer even before there are symptoms. A health worker like a nurse or doctor who has gotten training can do a simple check-up for cancer. In this check-up, they look at the mouth of the uterus, paint some vinegar on it, and see if there are any changes that look like cancer.

1. If this check-up was available in your community, do you think women would be willing to go for the test?
   1. If yes, why? Even if they had no symptoms?
   2. If no, why? What about if they had symptoms? Would that make them more likely to go?
2. If such a check-up was available for cervical cancer in your community, do you think men would support their wives to go for the test?
   1. If yes, why? ***Probe: Even if they had no symptoms?***
   2. If no, why? Would husbands be more likely to support their wives to go for a test if she had symptoms? What other factors might convince them to support their wives to undergo this check-up?
3. If this check-up was available in your community, would leaders in the community encourage women to go for the check-up?
   1. How can leaders encourage women to go for the check-up?
   2. Which leaders would be best able to encourage women?
   3. What can be done to help convince leaders to encourage women?
   4. How would you convince women in your community to go for screening? What would you say?

Stigma

1. Do you personally know anyone with cancer? If not, let’s assume a female family member or neighbor is suffering with cancer:
2. What are/would be the reactions of other family members and the neighbors to a woman with cancer?
   1. Do they think less of her?
   2. Gossip or speak badly about the woman or her family?—what do they say? What words are used to describe the woman?
   3. Would relationships with the woman or her family change? If yes, how?
3. What do you think are the most common problems faced by women with cancer?
   1. *Probe: At home/ in the village or neighborhood/workplace?*
   2. *Probe: What types of problems do women with cancer face? ( socially discriminated/not able to access credit/lose customers for a business*)
4. Do you think women who have cancer are isolated by society?
   1. If so, why?
5. Do you think people talk about cancer freely and openly in your society with one another?
   1. What about cancers specific to women, like cervical or breast cancer?
      1. ***If there is a difference, probe: why is there a difference?***
6. Do you think the family feels ashamed/ embarrassed to tell others if a woman in their family is suffering from cancer? *(Probe: Does it affect the relationships with family members)*
   1. What about cancers specific to women, like cervical or breast cancer?
      1. ***If there is a difference, probe: why is there a difference?)***
7. Do people feel uncomfortable being around a woman with cancer?  *(Probe: Sharing stuff/living with a cancer patient/ living close to a cancer patient/ working with a cancer patient, sitting or eating next to a cancer patient*).
   1. If so, why?
   2. Do people worry about "catching" it from another person?
8. Are there differences in the way women with cancer are viewed and treated by their families and the community if they have ‘female’ cancers, that is of the breast, uterus or mouth of the uterus compared to if they had a non-female organ cancer (e.g. brain, lung, blood)?
   1. If yes, why?

I have asked you all the questions I wanted to. Thank you for your time and patience. Do you have any questions for me?

# In-depth Interview Guide: Health Care Workers

Hello. My name is ___________, and I work for an American organization (Research Triangle Institute, International). As you know, we are doing a study in Karnataka state on health problems affecting women who are between the ages of 30 and 60 years. The objective of this discussion is to understand awareness of cancer and especially cancers that affect women, and access to and use of health services related to cancers that affect women in your community.

The interview will take about 30-45 minutes. Your participation is voluntary, and if you are not comfortable with a question you may choose not to answer it. There are no right or wrong answers. All comments, both positive and negative are welcome. We would also like to tape this interview so we make sure that we do not miss anything, and can write it down at a later time. When we write it down, we will not write down your personal information so there will be no way to identify what you have said after the contents of the tape have been written into notes. The tapes will remain in a locked cabinet and only the study staff will have access to the tapes and transcripts. After the analysis is completed all tapes will be destroyed.

**Introduction**: First, I would like to get some background information about you like how old you are, etc. *[Fill the Socio-demographic Form.]*

| Age | Education | Marital Status | Occupation/Role in Community |
| --- | --- | --- | --- |
|  |  |  |  |

Awareness about cervical cancer

1. What do you think are the most common cancers among Indian women?
2. What do you know about “Cervical Cancer?” ***If Health Worker not aware***, clarify, what do you know about cancer of the mouth of the uterus?
   1. How did you get this information?
      1. ***If not specified, PROBE:*** *medical or nursing school, on the job training, etc.*
   2. ***If has not heard of cervical cancer: read the following text to explain:*** “Our body is made of cells. Cancer is a disease in which these cells don’t work well, grow fast, and produce a lot of tissue. Cervical cancer is when this kind of growth happens on the mouth of the uterus – also called the cervix.”
3. Which women do you think are at greatest risk of getting Cervical Cancer) ***(Probe:*** *about different age groups, specific groups of women*)
   1. Why are these women at greatest risk?
4. Do you think women in your family and community are at risk of cervical cancer?
   1. If yes, why?
   2. If no, why not?
5. Do you think women can do things to avoid getting cervical cancer?  If so, what can they do?
6. Is there anything a woman can do to find cervical cancer early?
7. Do you think cervical cancer can be treated?

**For health workers who are not aware of cervical cancer ask the following:**

Facilitators and Barriers to uterus (pelvic) exams

Now I would like to ask you about uterus exams for women.

1. Do you women in your community get uterus exams?
   1. If yes, why do women in your community go for uterus exams?
      1. If not mentioned, *PROBE: pregnancy, symptoms (what kind of symptoms), knows someone who has been for exam, husband encourages, family member recommends, health care worker encouraged it, want to protect reproductive health or health in general to maintain family responsibilities, fears may have cancer.*
   2. If no, why not?
      1. ***If these issues do not come up, Probe on these issues***: *husband not supportive, family not supportive, distance, cost, does not feel comfortable, exam room is not private, fear health provider will share results (or fact that woman has come to exam) with others (not keep confidentiality), fear of exam, fear of cancer*
2. Do men in your community support and encourage their wives to go for uterus exams?
   1. If yes, why?
      1. What kind of support do they provide? ***(Probe*** *to get all possible types of support, e.g. encouragement, transport, money)*
   2. If no, why not?
3. Do you think women would be willing to go for a uterus exam as part of a routine/regular health check-up, even if they are not having any problems (symptoms)?
   1. If yes, why?
   2. If no, why not?
4. Do you think men would support their wives to go for a uterus exam as part of a routine/regular health check-up, even if the wife is not having any problems (symptoms)?
   1. If yes, why?
   2. If no, why not?
5. What conditions would make men more supportive of their wives going for a uterus exam?
   1. ***If not discussed, PROBE for:*** *female doctors, free screening, more convenient locations, comfortable place to be examined, place to be examined that is completely private (privacy), confidence that health provider will not share any information with others about the woman coming for a test or the results (confidentiality)*
6. In your role as a ______________ (e.g., ASHA, auxiliary nurse midwife, lady health visitor, nurse.):
   1. Do you encourage women to go for uterus exams?
      1. If yes, how do you encourage women?
      2. If no, why not? What would enable you to encourage women?
   2. Do you support women to go for a uterus exam?
      1. If yes, what kind of support do you provide?
      2. If no, why not? What would enable you to support women?
7. In your community, if a woman has problems like bleeding or abdominal pain, who does she talk to about those problems? (***Probe:*** *mothers-in-law, husband, health worker, neighbors****)***
   1. If the answer is no one, then probe: why do women not talk about these problems?
8. Do you think women feel comfortable talking about problems like bleeding or abdominal pain with health workers like you?
   1. Why? Why not?
   2. What about their husbands or other family members? Anyone else women feel comfortable talking to about such problems?
9. Do you feel comfortable talking about women’s health problems like bleeding or abdominal pain with women and men in your community?
   1. Why? Why not?
10. In your community, if a woman had problems like bleeding or abdominal pain, how would her husband or other family members respond? ***Probe***: would they support her to go to a health care provider?
11. What challenges have you faced in encouraging women to seek health care if they are experiencing problems such as bleeding or abdominal pain?
12. Have you undergone a uterus exam?

Check-up for cervical cancer

***Read explanatory text***: “As I explained earlier, cancer is when the cells in a part of the body grow fast and produce a lot of extra tissue. In women, the most common cancer affects the mouth of the uterus. There are several ways in which a health worker can detect cervical cancer even before there are symptoms. A health worker like a nurse or doctor who has gotten training can do a simple check-up for cancer. In this check-up, they look at the mouth of the uterus, paint some vinegar on it, and see if there are any changes that look like cancer. Now I am going to ask you a few questions about this check-up.

1. If this check-up was available in your community, do you think women would be willing to go for the test?
   1. If yes, why? Even if they had no symptoms?
   2. If no, why? What about if they had symptoms? Would that make them more likely to go?
2. If such a check-up was available for cervical cancer in your community, do you think men would support their wives to go for the test?
   1. If yes, why? ***Probe: Even if they had no symptoms?***
   2. If no, why? Would husbands be more likely to support their wives to go for a test if she had symptoms? What other factors might convince them to support their wives to undergo this check-up?
3. If this check-up was available in your community, would leaders in the community encourage women to go for the check-up?
   1. Which leaders would encourage women?
   2. How would you convince women in your community to go for screening? What would you say?
4. If this check-up was available in your community, would health workers like you encourage women to go for the check-up?
   1. Which health workers would be best able to encourage women?
   2. How would you convince women in your community to go for screening? What would you say?
   3. Have you undergone such a check-up yourself?

**For health workers who are aware of cervical cancer ask the following:**

Cervical cancer screening

***Read explanatory text***: Now I would like to ask you a few questions about screening women and detecting cervical cancer.

1. Do you know of different ways in which cervical cancer can be detected even before there are symptoms? ***If no, go to section above on “Check-up for Cervical Cancer”.***
   1. If yes, can you describe them?
2. If screening for cervical cancer was available in your community, do you think women would use the service?
   1. If yes, why? Even if they had no symptoms?
   2. If no, why? What about if they had symptoms? Would that make them more likely to go?
3. If screening for cervical cancer was available in your community, do you think men would support their wives to go for the test?
   1. If yes, why? ***Probe: Even if they had no symptoms?***
   2. If no, why? Would husbands be more likely to support their wives to go for a test if she had symptoms? What other factors might convince them to support their wives to undergo this check-up?
4. Do you think health workers like you would be willing to promote screening for cervical cancer?
   1. If yes, why? *Probe: common problem; can be treated; etc.*
   2. If no, why? *Probe: infrastructural or other health systems challenges; no time; too expensive to treat – or no treatment available, etc.*
   3. Which health workers would be best able to encourage women to go for this check-up?
5. What do you see as your role, if any, in promoting cervical cancer screening?
   1. What would you need in order to promote cervical cancer screening in your community?
   2. How would you convince women to go for screening? How would you convince their husbands to support their wives to undergo screening (or would you?)? What would you say?
6. Have you undergone this check-up?

Stigma

1. Let’s assume a woman in the community you work in is suffering with cancer. What are/would be the reactions of family members and neighbors to this woman with cancer?
   1. Do they think less of her?
   2. Gossip or speak badly about the woman or her family?
   3. Worry that she will die?
   4. What do they say?
   5. What words are used to describe the woman?
2. What do you think are the most common problems faced by women with cancer in the community where you work?
   1. *Probe: At home/ in the village or neighborhood/workplace?*
   2. *Probe: What types of problems do women with cancer face? ( socially discriminated/not able to access credit/lose customers for a business*)
3. Do you think women who have cancer are isolated by society?
   1. If so, why?
4. Do you think people talk about cancer freely and openly in your society with one another?
   1. What about cancers specific to women, like cervical or breast cancer?
      1. ***If there is a difference, probe: why is there a difference?***
5. Do you think the family feels ashamed/ embarrassed to tell others if a woman in their family is suffering from cancer? *(Probe: Does it affect the relationships with family members)*
   1. What about cancers specific to women, like cervical or breast cancer?
      1. ***If there is a difference, probe: why is there a difference?)***
6. Do people feel uncomfortable being around a woman with cancer?  *(Probe: Sharing stuff/living with a cancer patient/ living close to a cancer patient/ working with a cancer patient, sitting or eating next to a cancer patient*).
   1. If so, why?
   2. Do people worry about "catching" it from another person?
7. Are there differences in the way women with cancer are viewed and treated by their families and the community if they have ‘female’ cancers, that is of the breast, uterus or mouth of the uterus compared to if they had a non-female organ cancer (e.g. brain, lung, blood)?
   1. If yes, why?

I have asked you all the questions I wanted to. Thank you for your time and patience. Do you have any questions for me?

# In-depth Interview Guide: Women who have undergone cervical cancer screening

My name is _________ and I work for an American organization (Research Triangle Institution, International as a researcher for a study on cervical cancer screening. Our study aims to understand women’s experiences with cervical cancer screening. The findings from this study will be used to identify ways in which cervical cancer screening services may be improved.

The interview will take about one hour. Your participation is voluntary, and if you are not comfortable with a question you may choose not to answer it. There are no right or wrong answers. All comments, both positive and negative are welcome. We would also like to tape this interview so we make sure that we do not miss anything, and can write it down at a later time. When we write it down, we will not write down your personal information so there will be no way to identify what you have said after the contents of the tape have been written into notes. The tapes will remain in a locked cabinet and only the study staff will have access to the tapes and transcripts. After the analysis is completed all tapes will be destroyed.

The objective of this interview is to understand the experiences of women who have undergone cervical cancer screening. We will be asking some general information about you and then about your why you decided to go for screening, your experiences with the screening process, and your ideas about how to encourage other women to also get screened.

**Introduction**: First, I would like to get some background information about you like how old you are, etc. *[Fill the Sociodemographic Form.]*

**Trajectory for getting to cervical cancer screening**: Now, I would like to explore your experiences and understanding about your health, including how you went to get screening for cervical cancer.

1. ***Knowledge/awareness pre-screening***: I would like to begin by learning more about the time before you went for screening for cervical cancer and what you knew about cervical cancer at that time.
   1. Had you heard about cervical cancer at that time? If so, can you tell me what you knew?
   2. How did you hear about cervical cancer? Probe: From whom? (e.g. family member, doctor, friend)
   3. Had you heard about cervical cancer screening? What did you hear?
   4. How did you hear about cervical cancer screening? Probe: From whom? (e.g. family member, doctor, friend)
2. ***Knowledge/awareness*** ***currently (post screening).***
   1. Can you tell us what are the risk factors or causes of Cervical Cancer?

(Probe: Role of diet, life style, family history, any misconceptions – cancer is infectious, caused by supernatural causes, karmic fruits etc.)

- 1. Are you aware of any symptoms of cervical cancer?
     1. If, so what are those?
  2. Who told you about these symptoms? [If specific people or other sources (e.g. pamphlet, radio, TV) not provided, Probe: Who specifically told you about these symptoms? (family member, healthcare worker, neighbor, media, other?)]
     1. ***Probe (if not mentioned):*** did you learn about these when you went for screening?
  3. Which women do you think are at greatest risk of getting Cervical Cancer (Probe: Specifically about different age groups, specific groups of women)
     1. Why are these women at greatest risk?
  4. Do you think women in your family and communities are at risk of Cervical Cancer?
     1. If yes, why?
     2. If not, why not?
  5. Do you think women can do things to avoid getting Cervical cancer?  If so, what can they do?

1. ***Getting to the screening***: I would like to learn a little more about where, how and why you went for screening.
   1. Where did you get screened? In a clinic or other health facility or in a camp/as part of a campaign?
   2. Why did you go for screening? Probe:
      1. Did anyone encourage you to go? (e.g. husband, mother-in-law, friend, neighbor, health worker)
      2. Did you discuss it with anyone before you went? If so, who did you discuss it with? What did they say?
      3. ***If husband is not mentioned, probe***: What about your husband? Did you talk with him? If yes, what did he say? If no, why not?
   3. How did the decision about whether to get the screening get made?
      1. Who made the decision?
      2. Who was involved in making the decision? Were there any conflicts or disagreements regarding seeking screening? [Probe: can you describe those conflicts, tensions or disagreements? Any associated physical or psychological (denial of resources, humiliation, verbal abuse) violence?
      3. What factors influenced the decision? Probe if not discussed:
         1. symptoms (what kind of symptoms), knows someone who has been for exam, husband encourages, family member recommended, health care worker encouraged it, wanted to protect reproductive health or health in general to maintain family responsibilities, heard about it through media or campaign.
   4. Did you receive support from anyone to go for the screening? Probe: transport, funds, encouragement.
      1. Who provided support?
      2. Did anyone accompany you? If yes, who?

- 1. **ONLY ASK THIS** if the woman says the reason she went for screening is she had some kind of symptoms.
     1. What were the first signs or symptoms that you noticed?
     2. How did you feel about these signs/symptoms? *[Probe recognition as related to cancer, perceived seriousness, worry/anxiety, and coping strategies.]*
     3. What did you do about these signs/symptoms?
     4. Did you talk to anyone about it?
     5. If you spoke to someone about these signs/symptoms, how did they react? What did they recommend? *[Probe for each person they may have spoken to.]*
     6. *How long had you had symptoms before you went for screening?*

1. ***Experience with screening***: I would like to talk to you now about your experience when you went for screening.
   1. Where did you go for screening? *[Probe type of health facility (private or public? Level?); type of health care provider (physician, nurse, male/female provider? etc)]*
   2. Please describe the facility. Did you feel comfortable while you were waiting to be called in for the screening test?
      1. Did you know other women who were also waiting? If yes, how did that make you feel? Happy to know someone else who was waiting, or anxious that they now know you had screening?
   3. Can you describe how the screening exam was done? Did you understand what was happening? Did the health provider explain what they were doing?
   4. Did your knowledge about cervical cancer and cervical cancer screening increase after you were screened?
      1. What did you learn?
   5. What did you like about the facility (or camp) where the screening took place? [*Probe: female doctors, short wait times, close by home/not far, no or low cost, comfort, privacy, quality of providers, confidentiality]*
   6. What did you not like?
   7. Were you satisfied with the care you received overall? What could they have done differently to improve your experience?
   8. Any other feedback on how the process of screening for cervical cancer can be improved?
   9. What advice would you give to other women who are thinking about going for screening?
   10. What conditions would make it easier for you and other women to go for cervical cancer screening? Probe (female doctors, free screening, more convenient locations, privacy, confidentiality)
2. ***Post-Screening***
3. Have you talked about your screening experience with members of your family?
   - 1. Whom? What did you tell them?
   1. Have you talked about your screening experience with other people, besides family members?
      1. Whom? What did you tell them?
   2. Are there individuals (inside or outside your family) that you’ve consciously not shared this information with? *[Probe: why?]*
   3. How would you convince a family member or neighbor to go for screening? What would you say?
4. ***Stigma***:
5. Do you personally know anyone with cancer? If not, let’s assume a female family member or neighbor is suffering with cancer:
6. What are/would be the reactions of other family members and the neighbors to a woman with cancer?
   1. Do they think less of her?
   2. Gossip or speak badly about the woman or her family?—what do they say? What words are used to describe the woman?
   3. Would relationships with the woman or her family change? If yes, how?
7. What do you think are the most common problems faced by women with cancer?
   1. *Probe: At home/ in the village or neighborhood/workplace*
   2. *Probe: what specifically happens to her in the village/neighborhood/workplace? (e.g. socially discriminated, loses job, loses customers, cannot get loans, excluded from village groups, e.g. microfinance groups*)
8. Do you think women who have cancer are isolated by society?
   1. If so, why?
9. Do you think people talk about cancer freely and openly in your society with one another?
   1. What about cancers specific to women, like cervical or breast cancer? (***If there is a difference, PROBE: why do you think there is a difference?)***
10. Do you think the family feels ashamed/ embarrassed to tell others if someone in their family is suffering from cancer? *(****Probe: Does it affect the relationships with family members)***
    1. What about cancers specific to women, like cervical or breast cancer? ***(If there is a difference, PROBE: why do you think there is a difference?)***
11. Do people feel uncomfortable being around a woman with cancer?  ***(Probe if not mentioned: Sharing stuff/living with a cancer patient/ living close to a cancer patient/ working with a cancer patient, sitting or eating next to a cancer patient).***
    1. If so, why?
    2. Do people worry about "catching" cancer from another person?
12. Are there differences in the way women with cancer are viewed and treated by their families and the community if they have ‘female’ cancers, that is of the breast, uterus or mouth of the uterus compared to if they had a non-female organ cancer (e.g. brain, lung, blood)?
    1. If yes, why?

I have asked you all the questions I wanted to. Thank you for your time and patience. Do you have any questions for me?
